# The Role of the Rodent Lateral Orbitofrontal Cortex in Simple Pavlovian Cue-Outcome Learning Depends on Training Experience

**DOI:** 10.1093/texcom/tgab010

**Published:** 2021-02-09

**Authors:** Marios C Panayi, Simon Killcross

**Affiliations:** 1 School of Psychology, UNSW Sydney, Sydney, NSW 2052, Australia; 2 National Institute on Drug Abuse Intramural Research Program, Cellular Neurobiology Research Branch, Behavioral Neurophysiology Research Section, 251 Bayview Blvd., Baltimore, MD 21224, USA

**Keywords:** acquisition, flexible behavior, orbital prefrontal, Pavlovian, value

## Abstract

The orbitofrontal cortex (OFC) is a critical structure in the flexible control of value-based behaviors. OFC dysfunction is typically only detected when task or environmental contingencies change, against a backdrop of apparently intact initial acquisition and behavior. While intact acquisition following OFC lesions in simple Pavlovian cue-outcome conditioning is often predicted by models of OFC function, this predicted null effect has not been thoroughly investigated. Here, we test the effects of lesions and temporary muscimol inactivation of the rodent lateral OFC on the acquisition of a simple single cue-outcome relationship. Surprisingly, pretraining lesions significantly enhanced acquisition after overtraining, whereas post-training lesions and inactivation significantly impaired acquisition. This impaired acquisition to the cue reflects a disruption of behavioral control and not learning since the cue could also act as an effective blocking stimulus in an associative blocking procedure. These findings suggest that even simple cue-outcome representations acquired in the absence of OFC function are impoverished. Therefore, while OFC function is often associated with flexible behavioral control in complex environments, it is also involved in very simple Pavlovian acquisition where complex cue-outcome relationships are irrelevant to task performance.

## Introduction

The orbitofrontal cortex (OFC) is critical to behavioral flexibility when learning and behavior need to be updated to reflect a change in the environment (Kringelbach 2005; Klein-Flugge et al. 2013; Rudebeck and Murray 2014; Murray and Rudebeck 2018; Gardner et al. 2019). In particular, the OFC is necessary for appropriately updating behavior when the contingencies between predictive cues and outcomes change, or when outcomes change in value (Pickens et al. 2005; Walton et al. 2011; Panayi and Killcross 2018). The information encoded in OFC about the expected value and identity of predicted outcomes is necessary for flexibly updating behavior when these outcome features change. Population and single-unit neuronal firing in the OFC encodes many features of reward outcomes (e.g., size, preference, identity, time, location, probability, certainty, salience; Delamater 2007; Padoa-Schioppa 2009; Ogawa et al. 2013; Takahashi et al. 2013; Stalnaker et al. 2014; Sadacca et al. 2018; Zhou et al. 2019); furthermore, the coding of these features develops over the course of learning to predictive cues in anticipation of the expected outcome (Schoenbaum et al. 2009). There is also substantial evidence to suggest that this outcome expectancy information in the OFC is incorporated into midbrain dopaminergic reward prediction errors (Takahashi et al. 2011), which are critical for learning (Schultz 1998; Steinberg et al. 2013).

However, despite these close ties to the learning process, the OFC is typically not necessary for initial learning (Delamater 2007; Murray et al. 2007; Rudebeck and Murray 2014; Stalnaker et al. 2015; Izquierdo 2017), except in the most complex of circumstances (e.g., Walton et al. 2011). Lesions and functional inactivation of the OFC do not appear to disturb initial learning about Pavlovian cue-outcome relationships in a range of tasks, and instead only reveal their effects when the cue-outcome relationships change, or when the value of expected outcomes change, such as in reversal learning and outcome devaluation procedures (Butter 1969; Iversen and Mishkin 1970; Dias et al. 1996; Gallagher et al. 1999; Schoenbaum et al. 2003; West et al. 2011). To account for these effects, one class of OFC theories suggests that the OFC is necessary for representing information about the sensory-specific properties or identity of expected outcomes (Delamater 2007; Burke et al. 2008; Schoenbaum et al. 2009, 2011). A second, but complementary class of theories using a reinforcement learning framework suggests that the OFC is necessary for the representation of latent state information (Wilson et al. 2014). In reinforcement learning models, tasks such as Pavlovian conditioning can be divided into discrete physically observable states, such as “cue on,” “cue off,” and “reward,” and underlying latent states signaled by partially observable information recalled into working memory such as reinforcement history.

Both theories, while couched in different computational and theoretical frameworks, suggest similar roles for the OFC. Latent states encompass specific outcome expectancies and include a broader category of potential stimuli (e.g., internal context; Niv 2019). Implicit in these theories is that initial acquisition should be affected by OFC dysfunction if performance depends on specific outcome expectancy or latent states (e.g., the differential outcomes effect; McDannald et al. 2005; Boulougouris et al. 2007; Boulougouris and Robbins 2009; complex multiple-choice probabilistic learning tasks; Walton et al. 2011), but not in putatively “simple” single CS–US (cue-outcome) learning tasks (Gallagher et al. 1999), where the outcome identity and value of the US stays constant and is reliably predicted by the CS. While this null effect is often reported in procedures involving learning about multiple CSs and/or USs (Burke et al. 2008; Schoenbaum et al. 2009; Panayi and Killcross 2018), there is little evidence from tasks involving only a single CS–US relationship where a null result is clearly predicted. For example, Gallagher et al. (1999) found no effect of complete OFC lesions on single CS–US acquisition but stopped training before behavior reached asymptote (Schoenbaum et al. 2003).

Both latent state and sensory-specific outcome expectancy theories of OFC function predict a null effect of OFC lesions on initial acquisition learning, particularly in situations involving only a single CS–US relationship. Indeed, this null effect is often reported as an important feature of OFC dysfunction as it demonstrates that behavior can appear normal when the impoverished aspects of the underlying task representation are not directly relevant to task performance (Murray et al. 2007; Schoenbaum et al. 2009; Wilson et al. 2014; Stalnaker et al. 2015). Here, we directly tested this prediction in rats trained on a single CS–US Pavlovian task following lesions targeting the lateral OFC. Surprisingly, pretraining OFC lesions significantly increased Pavlovian acquisition behavior after extended training. In contrast, post-training lesions and intra-OFC infusions of muscimol impaired Pavlovian acquisition behavior. Using an associative blocking design, we confirmed that even though behavior was impaired, the underlying learning about the CS–US contingency remained intact.

## Methods and Materials

### General

#### Animals

Subjects were male Long Evans rats (Monash Animal Services, Gippsland, Victoria, Australia) approximately 4 months old. Rats were housed 4 per cage in ventilated Plexiglass cages in a temperature regulated (22 ± 1°C) and light regulated (12 h light/dark cycle, lights on at 7:00 AM) colony room. At least 1 week prior to behavioral testing, feeding was restricted to ensure that weight was approximately 95% of ad libitum feeding weight, and never dropped below 85%. All animal research was carried out in accordance with the National Institute of Health Guide for the Care and Use of Laboratories Animals (NIH publications No. 80-23, revised 1996) and approved by the University of New South Wales Animal Care and Ethics Committee.

#### Apparatus

Behavioral testing was conducted in 8 identical operant chambers (30.5 x 32.5 x 29.5 cm; Med Associates) individually housed within ventilated sound attenuating cabinets. Each chamber was fitted with a 3-W house light that was centrally located at the top of the left-hand wall. Food pellets could be delivered into a recessed magazine, centrally located at the bottom of the right-hand wall. Delivery of up to two separate liquid rewards via rubber tubing into the magazine was achieved using peristaltic pumps located above the testing chamber. The top of the magazine contained a white LED light that could serve as a visual stimulus. Access to the magazine was measured by infrared detectors at the mouth of the recess. Two retractable levers were located on either side of the magazine on the right-hand wall. A speaker located to the right of the house light could provide auditory stimuli to the chamber. In addition, a 5-Hz train of clicks produced by a heavy-duty relay placed outside the chamber at the back-right corner of the cabinet was used as an auditory stimulus. The chambers were wiped down with ethanol (80% v/v) between each session. A computer equipped with Med-PC software (Med Associates Inc.) was used to control the experimental procedures and record data.

#### Consumption Chambers

To provide individual access to reinforcers during the satiety and devaluation procedures, rats were placed into an individual cage (33 x 18 x 14 cm clear Perspex cage with a wireframe top). Pellet reinforcers were presented in small glass ramekins inside the box and liquid reinforcers were presented in water bottles with a sipper tube. One day prior to the target procedure, all rats were exposed to the individual cages and given 30 min of free access to home cage food and water to reduce novelty to the context and consuming from the ramekin and water bottles.

#### Locomotor Activity

Locomotor activity was assessed in 8 identical boxes measuring 50 x 36 x 18 cm (length x width x height), housed in a sound attenuated room. Each box consisted of 4 opaque white polyurethane walls and floor and a removable roof. In the center of the roof was an 18 x 40 cm grid of 3 x 3 mm ventilation holes. Two custom pairs of infrared beam detectors spanned the width of the box to detect locomotor activity and were located 15 cm from each end of the box. Beam breaks, corresponding to activity within the box, were recorded on a computer equipped with Med-PC software (Med Associates Inc.).

#### Surgery

Excitotoxic lesions targeting the lateral OFC were performed in Experiment 1 and Supplementary Experiment 1. Rats were anesthetized with isoflurane, their heads shaved, and placed in a stereotaxic frame (World Precision Instruments, Inc.). The scalp was incised, and the skull exposed and adjusted to flat skull position. Two small holes were drilled into the skull and the dura mater was severed to reveal the underlying cortical parenchyma. A 1-μL Hamilton needle (Hamilton Company) was lowered through the two holes targeting the lateral OFC (co-ordinates specified below). Stereotaxic co-ordinates were AP: +3.5 mm; ML: ±2.2 mm; D-V: −5.0 mm from bregma. At each site, the needle was first left to rest for 1 min. Then, an infusion of N-methyl-D-aspartic acid (NMDA; Sigma-Aldrich, Switzerland), dissolved in phosphate buffered saline (pH 7.4) to achieve a concentration of 10 μg/μL, was infused for 3 min at a rate of 0.1 μ/min. Finally, the needle was left in situ for a further 4 min to allow the solution to diffuse into the tissue. Following the diffusion period, the needle was retracted, and the scalp cleaned and sutured. Sham lesions proceeded identically to excitotoxic lesions except that during the infusion period no infusion occurred. After a minimum of 1 week of postoperative recovery, rats were returned to food restriction for 2 days prior to further training.

In Experiments 2, 3, and Supplementary Experiment 2, bilateral guide cannulae were surgically implanted targeting the lateral OFC. Rats were anesthetized with isoflurane, their heads shaved, and placed in a stereotaxic frame (World Precision Instruments, Inc.). The scalp was incised, and the skull exposed and adjusted to flat skull position. Two small holes were drilled for the cannulae using a high-speed drill, and four holes were hand drilled on different bone plates to hold fixing screws. Bilateral stainless-steel guide cannulae (26-gauge, length 5 mm below pedestal; Plastics One, Roanoke, VA) were lowered into the lateral OFC (AP: +3.5 mm; ML: ±2.2 mm; D-V: −4.0 mm from bregma). Cannulae were held in place by dental cement and anchored to the skull with 4 fixing screws. Removable dummy cannulae were inserted into the guide cannulae to prevent them from blocking. After 1 week of postoperative recovery, rats were returned to food restriction for 2 days prior to further testing.

#### Drugs and Infusions

The GABA_A_ agonist muscimol (Sigma-Aldrich, Switzerland) was dissolved in 0.9% (w/v) nonpyrogenic saline to obtain a final concentration of 0.5 μg/0.5 μL. Nonpyrogenic saline 0.9% (w/v) was used as the saline control. During infusions, muscimol or saline was infused bilaterally into the lateral OFC by inserting a 33-gauge internal cannula into the guide cannula that extended 1 mm ventral to the guide tip. The internal cannula was connected to a 25 μL glass syringe (Hamilton Company) attached to a microinfusion pump (World Precision Instruments, Inc.). A total volume of 0.5 μL was delivered to each side at a rate of 0.25 μL/min. The internal cannula remained in place for an additional 1 min after the infusion and then removed. During the infusion, procedure animals could move freely in a bucket to minimize stress. Dummy cannulae were removed prior to, and replaced immediately after, infusions. For the two training sessions prior to infusions, all animals received dummy infusions which were identical to the infusion procedure, except that no liquids were infused. These dummy infusions were performed to familiarize the rats with the microinfusion procedure and thereby minimize stress. Dummy infusions were also conducted on test sessions after the infusions to minimize differences in handling between experimental stages.

#### Reinforcers

The reinforcers used were a single grain pellet (45 mg dustless precision grain-based pellets; Bio-serv), a single sucrose pellet (45 mg dustless precision sucrose pellets; Bio-serv), and 20% w/v maltodextrin solution (Myopure, Petersham) flavored with 0.4% v/v concentrated lemon juice (Berri, Melbourne) to provide unique sensory properties to the reinforcer. Liquids were delivered over a period of 0.33 s via a peristaltic pump which corresponded to a volume of 0.2 mL. The volume and concentration of liquid reinforcers was chosen to match the calorific value of the corresponding grain and sucrose pellet reward and have been found to elicit similar rates of Pavlovian and instrumental responding as a pellet reward in other experiments conducted in this lab. In all experiments involving liquids, the magazine was scrubbed with warm water and thoroughly dried between sessions to remove residual traces of the liquid reinforcer. To reduce neophobia to the reinforcers, 1 day prior to magazine training sessions all animals were pre-exposed to the reinforcers (10 g of pellets per animal and 25 mL of liquid reinforcer per animal) in their home cage.

#### Magazine Training

All animals received one session of magazine training for each experimental reinforcer with the following parameters: reward delivery was on an RT60 s schedule for 16 rewards. When necessary, sessions were separated by at least 2 h and the order of reinforcer identity was counterbalanced between groups.

#### Behavior

CS responding was operationalized as the number of magazine entries during the CS period. PreCS responding was operationalized as the frequency of responding during the immediately preceding the CS period and was used as a measure of baseline responding to the testing context. PreCS responding was analyzed separately, and any group differences identified and reported. Data were presented as CS – PreCS difference scores, which reflect discriminative responding to the CS. All data were analyzed with mixed ANOVAs using R statistical software (Lenth et al. 2020; R Core Team 2020; Singmann et al. 2020), and significant interactions of interest were followed up with ANOVAs on the relevant subset of data, and simple effects with a Tukey family-wise error rate correction. Where relevant, planned linear and quadratic orthogonal trend contrasts and their interactions between groups were analyzed to assess differences in rates of responding.

## Experiment 1: Pretraining OFC Lesions

### Subjects

Subjects were forty-eight (*N* = 48) rats, tested in two cohorts. Cohort 1, *n* = 16 rats weighing between 280 and 361 g (*M* = 312.2 g) and cohort 2, *n* = 32 rats weighing between 271 and 328 g (*M* = 296.3 g).

### Training

#### Pavlovian Acquisition

Following magazine training, all rats received 21 sessions of Pavlovian acquisition training. Each session consisted of 16 presentations of a single auditory CS (a 15 s train of clicks) presented on a VT90s schedule (ranging from 60 to 120 s). A single pellet (US) was delivered at the termination of each CS. The session duration was 28 min and animals were left in the chamber for an additional 2 min before being removed. Animals received either 1 session per day, or 2 sessions per day separated by at least 2 h.

#### Subgroup 1: General Satiety Prefeeding

At the end of acquisition training on day 21, a subgroup of animals (sham *n* = 8, lesion *n* = 8) were taken off food restriction and given 24 h free access to their home cage food before further acquisition training on day 22. This session was rewarded as per acquisition training. At the end of day 22, animals were put back on food restriction and continued acquisition training.

#### Subgroup 2: Devaluation

Following initial Pavlovian acquisition of a single CS–US association, a second subgroup of animals (sham *n* = 8, lesion *n* = 8) were retrained with two novel unique CS–US associations intended to test devaluation in a taste aversion procedure.

#### Novel Acquisition

Novel acquisition of two unique CS–US associations was conducted with identical parameters to initial acquisition training, 2 sessions per day for 14 days. There were 16 trials per session separated by a vITI90s, with each trial consisting of a 15 s CS coterminating with reward. Unlike initial acquisition, the two CSs were an 80 dB white noise and a 2800 Hz, 80 dB tone followed by either a sucrose pellet or a lemon flavored maltodextrin liquid reinforcers (CS–US identities counterbalanced between animals).

#### Taste Aversion

Taste aversion took place in the devaluation chambers and involved 30 min exposure to one US every day, alternating each day for 4 days. Following fee access to a US, animals were immediately injected i.p. with either 0.15 M LiCl or 0.9% saline (15 mL/kg). The outcome paired with nausea induced by injection of LiCl was designated the devalued outcome and the outcome paired with neutral saline injections was designated the nondevalued outcome (counterbalanced between animals). Following the final day of injections, all animals were given a day of rest in their home cage to allow hunger levels to return to normal after taste aversion training.

#### Devaluation Test

Animals were tested with a single session of CS training except that no rewards were delivered, that is, in extinction. Data from the first trial were analyzed at test.

#### Locomotor Activity

At the end of the experimental procedures, all animals were assessed for locomotor activity over a 1-h period.

## Experiment 2: Post-Training Muscimol Inactivation

### Subjects

Subjects were 32 (total *N* = 32) male Long Evans rats (Monash Animal Services) approximately 4 months old, weighing between 285 and 350 g (*M* = 319.7 g).

#### Pavlovian Acquisition

Animals were given 9 sessions, 1 session per day, of Pavlovian acquisition training with session parameters identical to those described in Experiment 1. This number of sessions was chosen because the effect of pretraining lesions appeared after around 9 session in Experiment 1. Briefly, each session consisted of a VT90s ITI with 16 trials consisting of a 15 s click CS coterminating with a single pellet US. Following the final day of training, all animals were taken off food restriction and received surgical implantation of guide cannulae.

### Post-Training

#### Preinfusion

Following postoperative recovery animals were returned to food restriction for a day before receiving a further 2 days of acquisition training as per pretraining. However, immediately prior to entering the chamber all animals received a dummy infusion.

#### Infusion

Animals were pseudo-randomly assigned to one of two infusion groups such that performance was matched and there were no differences between groups on the final day of preinfusion acquisition. For the next 4 days, all animals received an infusion of saline or Muscimol immediately prior to entering the testing chamber for a Pavlovian acquisition session.

#### Postinfusion

On the final 2 days of training, all animals received a further 2 days of acquisition training immediately preceded by a dummy infusion.

## Supplementary Experiment 1: Post-Training OFC Lesions

### Methods

#### Subjects

Subjects were 24 (total *N* = 24) male Long Evans rats (Monash Animal Services) approximately 4 months old, weighing between 317 and 369 g (*M* = 338.9 g).

#### Prelesion Training

*Pavlovian acquisition*. All animals received 9 days of Pavlovian acquisition training, 1 session per day. On the final day of training, all animals were removed from food restriction for at least 24 h before receiving sham or excitotoxic lesions of the OFC. Lesion conditions were pseudorandomly assigned to animals such that group performance was matched on the final day of acquisition and an equal number of animals were assigned to each lesion condition in each homecage.

#### Postlesion Training

*Pavlovian acquisition*. Following postoperative recovery, all animals were returned to food restriction for 24 h before receiving an additional 9 days of acquisition training.

## Supplementary Experiment 2: OFC Inactivation Early in Acquisition

### Subjects

Subjects were 32 (total *N* = 16) male Long Evans rats (Monash Animal Services) approximately 4 months old, weighing between 321 and 399 g (*M* = 357.4 g).

### Surgery

Surgical implantation of cannulae occurred prior to any behavioral training.

#### Pavlovian Acquisition

Animals were given 10 sessions, 1 session per day. Briefly, each session consisted of a VI 200 s ITI with 16 trials consisting of a 10s light CS (illumination of the house light at the back of the chamber) coterminating with a single pellet US. Subjects received mock infusions on days 3 and 4, and either Saline or Muscimol was infused prior to entering the chamber on days 5–9. On day 10, all animals received a mock infusion.

## Experiment 3: OFC Inactivation Prior to Associative Blocking

### Subjects

Subjects were 32 (total *N* = 32) male Long Evans rats (Monash Animal Services) approximately 4 months old, weighing between 299 and 395 g (*M* = 331.5 g).

### Surgery

Surgical implantation of cannulae occurred prior to any behavioral training.

### Training

The design of the experiment was such that 4 CSs were designated as cues A, B, C, and D. Cues A and C were always visual cues, either darkness caused by extinguishing the houselight or flashing panel lights (5 Hz; Fig. 3*A*). Cues B and D were always auditory cues, either an 80 dB white noise or a 5 Hz train of clicks. Throughout all training sessions, the house light was always illuminated unless it was extinguished to act as a visual cue. All cues lasted 10 s and coterminated with the delivery of the US, 2 pellets delivered consecutively 0.25 s apart. The identity of the cues was counterbalanced between subjects except that A and C were always visual cues and B and D were always auditory cues. Simultaneous audio-visual compounds were designated as AB and CD. Pavlovian training sessions were always 56 mins long such that there were 16 trials with a vITI 200 s (range 100–300 s); animals were left in the chambers for an additional 2 min before being removed.

#### Food Restriction and Magazine Training

Magazine training sessions consisted of an RT120s reward delivery schedule for 16 rewards. Each reward consisted of 2 pellets delivered to the magazine 0.25 s apart.

#### Stage 1

Stage 1 acquisition involved 10 days of acquisition to cue A, 16 trials per session. On days 1–4 of training, all animals received dummy infusions to familiarize them to the infusion procedure. Animals were then split into two groups with matched performance on day 4. On days 5–10, all animals received an infusion of saline or muscimol immediately prior to entering the test chambers.

#### Pre-Exposure

On day 11, all rats received pre-exposure to auditory cues B and D, 4 nonrewarded presentations of each cue vITI 200 s. This was done to minimize novelty to the auditory cues during compound training in stage 2. All animals received dummy infusions prior to the session.

#### Stage 2

On days 12–14, all animals received stage 2 audio-visual compound training. Sessions involved 8 presentations of compound AB and 8 presentations of CD (pseudorandomly presented such that a compound was never repeated more than 2 times in a row). The compounds were rewarded with 2 pellets, the same US that was used in stage 1. All animals received dummy infusions prior to each session.

#### Test

On days 15 and 16, all animals were tested in extinction for responding to the target auditory cue B and the overshadowing control cue D (8 presentations of each cue, pseudorandom trial order, vITI 200 s). All animals received dummy infusions prior to each session.

#### Reacquisition

On days 17–19, all animals received reacquisition training to cue B (16 trials per session) to test for differences in rates of reacquisition to the blocked cue. On days 20–21, animals were tested for reacquisition to cue A (16 trials per session) to test for differences in the rate of reacquisition to the blocking cue.

### Data Availability

Raw data available at: Panayi MC. 1 February 2021. Data for: The role of the rodent lateral OFC in simple Pavlovian cue-outcome learning depends on training experience. doi: 10.17605/OSF.IO/TNBH7.

## Results

### Experiment 1: Pretraining OFC Lesions

#### Histology and Group Allocation

Lesion damage is depicted in Supplementary Figure S1 (supplement 1). Lesion extent was judged by a trained observer blind to group allocation. A lesion was retained if there was evidence of significant bilateral damage constrained to LO or DLO. Animals were excluded if there was only unilateral LO/DLO damage, evidence of damage to the dorsal part of the anterior olfactory nucleus ventral to LO/DLO or if there was extensive damage to the white matter of the forceps minor of the corpus callosum. One lesioned animal did not recover from surgery, four lesion animals had only unilateral OFC damage, and one lesioned animal had extensive white matter damage. Forty-two animals were retained (*N* = 42, sham *n* = 24, lesion *n* = 18), of which subgroup 1 contained 15 (*N* = 15; sham *n* = 8, lesion *n* = 7) and subgroup 2 contained 13 (*N* = 13; sham *n* = 8, lesion *n* = 5).

#### PreCS Analysis

Analysis of the PreCS period using a Group (sham, lesion) x Block (1–7) mixed ANOVA revealed that responding was significantly higher in the lesion group than the sham group (main effect of Group *F*_(1, 40)_ = 7.24, *P* = 0.01). Furthermore, while responding increased over blocks (main effect of Block *F*_(6, 240)_ = 20.37, *P* < 0.001; positive linear trend *F*_(1, 40)_ = 33.18, *P* < 0.001), this increase was greater in the lesion than the sham group (Block x Group interaction *F*_(6, 240)_ = 2.52, *P* = 0.02; linear trend interaction *F*_(1, 40)_ = 5.34, *P* = 0.03). During the first block, PreCS responding was similar between groups (Sham *M* = 2.07, SD = 0.60; Lesion *M* = 2.13, SD = 0.90), by the final block PreCS responding was higher in the Lesion group (*M* = 4.30, SD = 1.95) than the Sham group (*M* = 2.76, SD = 2.30).

#### Acquisition

**Figure 1:**
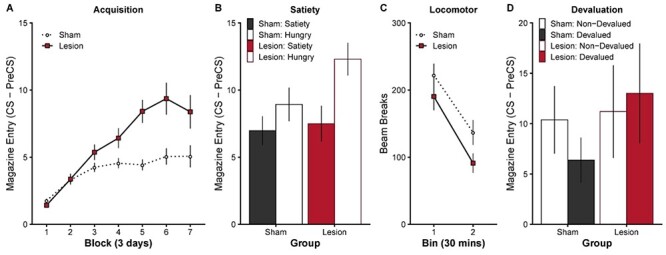
The effect of pretraining OFC lesions on the acquisition of simple Pavlovian cue-outcome relationship. Representative lesion damage and histology depicted in Supplementary Figure S1 (supplement 1). (*A*) Experiment 1: OFC lesions significantly enhance acquisition behavior to a simple Pavlovian cue (CS) predicting a food pellet. Responding during the baseline PreCS period is subtracted from responding during the CS period (i.e., CS–PreCS). Data presented in blocks of 3 days. (*B*) The effect of manipulating general levels of satiety (24 h ad libitum access to food) on Pavlovian acquisition behavior in a subset of rats (subgroup 1; sham *n* = 8, lesion *n* = 7). General satiety reduced behavior in the lesion group and abolished group differences (sated), which returned when tested hungry 24 h later. The effect of satiety was also evident on the first trial of the session Supplementary Figure S1 (supplement 2). (*C*) Locomotor activity (as reflected by infrared beam breaks in a novel open-field) measured over 1 h (separated into 30 min blocks) shows no significant hyperactivity in the OFC lesion group. (*D*) The effect of outcome-specific devaluation is abolished by OFC lesions (subgroup 2; sham *n* = 8, lesion *n* = 5). After retraining with two unique Pavlovian cues and outcomes (Supplementary Figure S1 [supplement 3A]), one outcome was paired with injections of LiCl to establish an outcome specific taste aversion (Supplementary Figure S1 [supplement 3B]). At test, responding to the cue that predicted the now Devalued outcome (vs. the nondevalued control outcome) revealed that the sham group appropriately reduced behavior for the devalued outcome, whereas the lesion group did not. Error bars depict ±SEM.

Pretraining OFC lesions significantly increased responding to the Pavlovian cue relative to sham control animals (Fig. 1*A*; lesions depicted in Supplementary Figure S1 [supplement 1]). Analysis of conditioned responding was conducted as a CS–PreCS difference score such that levels of responding reflected discriminative performance to the cue (CS) above baseline (PreCS). Acquisition of responding to the CS was significantly greater in the lesion group than the sham group (main effect of Group $F\big(1,40\big)=10.83$, $P=0.002$, Block $F\big(\mathrm{6,240}\big)=34.07$, $P<0.001$, and Group x Block interaction $F\big(\mathrm{6,240}\big)=7.33$, $P<0.001$). Follow up comparisons on each block revealed that responding in the lesion group was significantly higher than the sham group during the last 4 blocks (Block 1 $t(40)=-1.67$, $P=0.103$, Block 2 $t(40)=0.14$, $P=0.893$, Block 3 $t(40)=1.79$, $P=0.082$, Block 4 $t(40)=2.39$, $P=0.022$, Block 5 $t(40)=4.59$, $P<0.001$, Block 6 $t(40)=3.48$, $P=0.001$, Block 7 $t(40)=2.32$, $P=0.026$). Given the ubiquity of nonsignificant effects of OFC lesions on acquisition learning in the literature, two independent replications of this novel effect were conducted (combined here; same pattern of statistical significance in both independent replications) which confirmed the effect was robust.

#### Locomotor Activity

The enhanced responding observed during acquisition in the OFC lesion group could simply reflect an enhancement of general locomotor activity. However, locomotor activity (Fig. 1*C*) did not differ between groups (main effect of TimeBin $F\big(1,33\big)=62.93$, $P<0.001$, but no significant effect of Group $F\big(1,33\big)=2.87$, $P=0.100$, or Group x TimeBin interaction $F\big(1,33\big)=0.36$, $P=0.555$). Therefore, the enhanced responding during acquisition was not simply due to lateral OFC lesions inducing hyperactivity, consistent with previous findings (e.g., Lasseter et al. 2009; Panayi and Killcross 2018).

#### General Satiety

To test whether the enhanced responding following pretraining OFC lesions was sensitive to levels of hunger or shifts in general motivation, a subgroup of animals (subgroup 1) was tested when sated, that is, following 24 h ad libitum access to home-cage food (Fig. 1*B*). General satiety, did not affect the rate of responding in the sham group (Sham: Satiety vs. Hungry $t(13)=-1.38$, $P=0.191$) but significantly suppressed responding in the lesion group (Lesion: Satiety vs. Hungry $t(13)=-4.24$, $P=0.001$) compared with subsequent testing 24 h later when hungry again (no significant main effect of Group $F\big(1,13\big)=1.43$, $P=0.253$, but a significant main effect of Hunger $F\big(1,13\big)=16.30$, $P=0.001$, and Group x Hunger interaction $F\big(1,13\big)=4.63$, $P=0.051$). Since the satiety test session was rewarded, it is possible that OFC lesioned animals could learn that the reward was less valuable by direct experience with the reward, similar to incentive learning effects normally observed in instrumental conditioning (Dickinson and Balleine 2002). However, this possibility is unlikely as responding was comparable between groups on the first trial of the satiety test ($t(13)=1.04$, $P=0.317$, Supplementary Figure S1 [supplement 2]), before the first reward was delivered. This suggests that, consistent with previous reports (e.g., McDannald et al. 2005), animals with lateral OFC lesions are sensitive to shifts in hunger and general motivation.

#### Devaluation Test

OFC lesions have been shown to cause characteristic deficits in Pavlovian outcome devaluation (Gallagher et al. 1999; Pickens et al. 2003, 2005; Panayi and Killcross 2018). Therefore, to test whether the present lesion manipulation was comparable with other reports, we tested a subgroup of animals (subgroup 2) on Pavlovian outcome devaluation. First, the sham and lesion animals were given novel acquisition training of two novel and unique cue-outcome relationship (see Supplementary Figure S1 [supplement 3A]). A specific taste aversion was then established by pairing consumption of one of the outcomes with illness (i.p. injection of lithium chloride; Devalued), and the value of the other outcome was left intact (i.p. injection of saline; Nondevalued). Both groups learned the novel cue-outcome associations and acquired the specific taste aversion (see Supplementary Figure S1 [supplement 3B]).

Finally, during a devaluation test (Fig. 1*D*), the two cues were presented in extinction. The sham group showed a significant devaluation effect, that is, responding was lower to the devalued than nondevalued cue ($t(11)=-3.06$, $P=0.011$). In contrast, the devaluation effect was abolished in the lesion group, and responding remained high to both the devalued and nondevalued cue ($t(11)=1.09$, $P=0.300$; Significant Group x Cue interaction $F\big(1,11\big)=7.55$, $P=0.019$, but no main effect of Group $F\big(1,11\big)=0.54$, $P=0.479$, or Cue $F\big(1,11\big)=1.09$, $P=0.320$). This finding successfully replicates the finding that both complete OFC and focal lateral OFC lesions abolish the outcome devaluation effect in rodents (Gallagher et al. 1999; Pickens et al. 2003, 2005; Panayi and Killcross 2018).

### Experiment 2: Post-Training Muscimol Inactivation

#### Histology and Group Allocation

Cannulae placements are illustrated in Supplementary Figure S2 (supplement 2). One animal did not recover from surgery and was excluded. Three animals were excluded because of the cannulae assembly detaching from the skull. A further 3 animals were excluded because of failing to consume the pellets after recovery from surgery. One animal from the muscimol group was excluded from analysis because of a cannula tip embedded within the white matter of the forceps minor of the corpus callosum. Therefore, a total of 8 animals were excluded leaving *N* = 24 (saline *n* = 12, muscimol *n* = 12).

#### PreCS Rates

PreCS baseline responding did not differ between infusion groups across training. In particular, during the infusion period a Group x Day (4 days) mixed ANOVA on PreCS responses revealed a significant effect of Day (*F*_(3, 66)_ = 5.95, *P* = 0.001) but no significant effect of Group (*F*_(1, 22)_ = 0.01, *P* = 0.93) or Group x Day interaction (*F*_(3, 66)_ = 0.41, *P* = 0.741). PreCS response rates on these days were, saline *M* = 0.70, SD = 0.48, muscimol *M* = 0.72, SD = 0.48.

The enhanced Pavlovian responding observed following OFC lesions (Fig. 1*A*) may be due to enhanced learning of a general cue-outcome predictive relationship in the OFC lesion group (see Supplementary Figure S2 [supplement 1]). This is consistent with a role for the OFC in representing outcome expectancy information. For example, incremental learning about a cue-outcome relationship is thought to depend upon prediction errors (Rescorla and Wagner 1972; Mackintosh 1975; Pearce and Hall 1980; Sutton and Barto 1998; LePelley 2004; Esber and Haselgrove 2011; Nasser et al. 2017), that is, the difference between the experience outcome value and the expected outcome value. The expected outcome value of a cue is incrementally updated until this prediction error discrepancy is minimized. If the OFC carries some aspect of outcome expectancy information (Baxter et al. 2000; Pears et al. 2003; Schoenbaum et al. 2009; Takahashi et al. 2009, 2011), then OFC lesions might consistently reduce/underestimate the expected value of a cue which in turn would result in abnormally persistent prediction errors and enhanced learning. Therefore, disruption of OFC function should temporarily lower expected value, and enhance prediction errors and learning supported by other brain regions (for modeling of this prediction see Supplementary Figure S2 [supplement 1]). We tested this hypothesis by inactivating the OFC after first successfully acquiring cue-outcome learning, that is, when expected value is high and prediction errors are low. If the OFC carries some aspect of the learned expected value, then inactivation of the OFC should restore prediction errors at the time of reward and responding should increase to reflect new learning. Following this, returning function to the OFC should result in an overexpectation of the value of the outcome, and performance should decrease to reflect the extinction of this overexpectation. Importantly, while this account is couched in terms prediction-error learning mechanisms, the prediction remains true for any account of OFC lesions enhancing learning (see Supplementary Figure S2 [supplement 1]).

**Figure 2:**
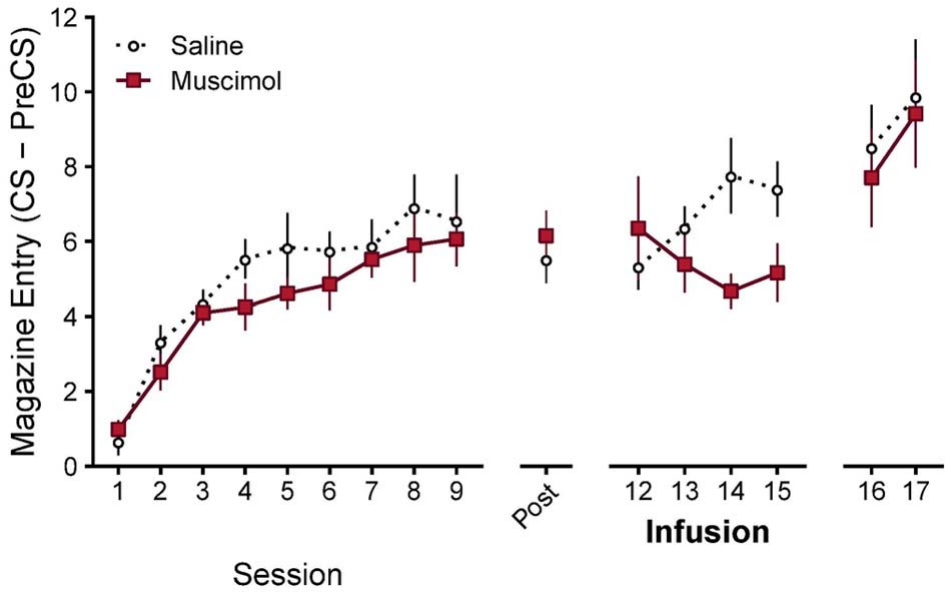
Post-training OFC inactivation suppresses Pavlovian acquisition behavior, in contrast to pretraining lesions which increased Pavlovian acquisition behavior. If pretraining lesions increase Pavlovian learning, then post-training lesions or inactivation should also increase learning (rationale and learning model predictions in Supplementary Figure S2 [supplement 1]). Experiment 2: Rates of discriminative responding (CS–PreCS) during initial acquisition (sessions 1–9), postoperative recovery (post), following intra-OFC infusion of muscimol or saline (sessions 12–15), and without infusion (sessions 16–17). Cannulae placements depicted in Supplementary Figure S2 (supplement 2). The effect of post-training lesions on acquisition revealed the same pattern of results (Supplementary Figure S2 (supplements 3 and 4). Error bars depict ±SEM.

We tested this hypothesis by first training a new group of animals on the same simple Pavlovian task for 9 days, before implantation of bilateral cannulae targeting the OFC (Fig. 2, days 1–9; significant main effect of day $F\big(\mathrm{8,176}\big)=25.42$, $P<0.001$, but no main effect of Group $F\big(1,22\big)=1.08$, $P=0.310$, or Group x Day interaction $F\big(\mathrm{8,176}\big)=0.54$, $P=0.825$). Following postoperative recovery (histology depicted in Supplementary Figure S2 [supplement 2]), and prior to infusion, response levels were similar in both groups (Fig. 2, post; no significant differences between Groups $t(22)=-0.68$, $P=0.501$).

Contrary to our prediction, intra-OFC muscimol infusions disrupted rather than enhanced further acquisition of responding relative to the saline group (Fig. 2, infusion–days 12–15; significant Group x Day interaction $F\big(3,66\big)=5.03$, $P=0.003$, but no main effect of Group $F\big(1,22\big)=1.90$, $P=0.182$, or Day $F\big(3,66\big)=0.32$, $P=0.809$). Simple effects revealed significantly greater responding in the saline group on the last 2 days of infusions (muscimol vs. saline: day 12 $t(22)=0.67$, $P=0.508$, day 13 $t(22)=-1.03$, $P=0.315$, day 14 $t(22)=-2.79$, $P=0.011$, day 15 $t(22)=-2.08$, $P=0.049$). Furthermore, the saline group increased responding across infusion days 12–15 (saline: significant positive linear trend $t(22)=2.79$, $P=0.011$), whereas the muscimol group did not (muscimol: no significant linear trend $t(22)=-1.57$, $P=0.131$). Therefore, post-training inactivation of the OFC impaired acquisition.

Postinfusion, with function returned to the OFC, the group differences observed under drug infusion were no longer apparent, and both groups continued to acquire responding at similar levels (Fig. 2, days 16–17; significant main effect of day $F\big(1,22\big)=16.05$, $P=0.001$, but no main effect of Group $F\big(1,22\big)=0.11$, $P=0.740$, or Group x Day interaction $F\big(1,22\big)=0.21$, $P=0.649$). Therefore, the effect of OFC inactivation did not persist, which suggests that the disruption in acquisition following OFC inactivation might not have impaired learning per se.

Furthermore, we tested post-training lesions to rule out the possibility that the differences between pre- and post-training OFC manipulations were simply due to differences in the method of manipulation, that is, excitotoxic lesions versus inactivation using a GABA-A agonist. Consistent with muscimol inactivation, post-training lesions significantly impaired Pavlovian acquisition (see Supplementary Experiment 1: Supplementary Figure S2 [supplements 3 and 4]). Therefore, it is unlikely that the difference between pre- and post-training OFC manipulations observed in Experiments 1 and 2 are due to the method of manipulation.

### Experiment 3: OFC Inactivation Prior to Associative Blocking

**Figure 3:**
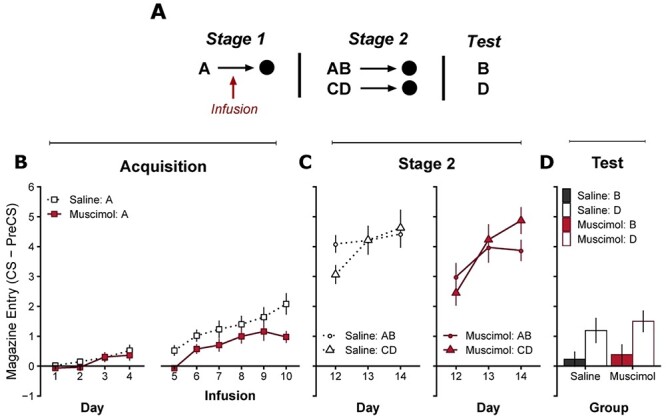
The effect of OFC inactivation during acquisition on subsequent learning in a Pavlovian blocking design. Experiment 3: (*A*) The design used to achieve blocking of learning to cue B during stage 2 by pretraining cue A in stage 1. OFC infusions of saline or muscimol were performed during stage 1 after the first 4 days of initial acquisition to cue A. Cues A and C were always visual cues, either darkness caused by extinguishing the houselight or flashing panel lights (5 Hz). Cues B and D were always auditory cues, either an 80 dB white noise or a 5 Hz train of clicks. All cues lasted 10 s, and reward was always a single food pellet. Cannulae placements depicted in Supplementary Figure S3 (supplement 3). (*B*) Pavlovian acquisition to cue A over 10 days, with intact OFC (days 1–4) and following infusion of saline or muscimol to functionally inactivate the OFC (days 5–10). Muscimol infusions significantly suppressed responding to cue A. (*C*) Performance during stage 2 of blocking to cue compounds AB and CD in the saline (left) and muscimol (right) infusion groups. A focused analysis of responding within day 12 is presented in Supplementary Figure S3 (supplement 4). (*D*) Responding during an extinction test to “blocked” cue B and the overshadowing control cue D. Supplementary Figure S3 (supplement 5) shows subsequent reacquisition to cues B and A to assess possible differences in attentional strategies between the saline and muscimol group. Significantly reduced responding to cue B relative to cue D indicates that learning about cue A effectively blocked subsequent learning to cue B in both the muscimol and saline groups. Pavlovian responding quantified by the rate of discriminative responding (CS–PreCS). Error bars depict ±SEM.

OFC inactivation during acquisition suppressed cue responding, but it is unclear if this reduction in behavior is due to suppression of additional learning or behavioral performance (Fig. 2). This ambiguity is predominantly driven by the assumption that an animal’s response levels represent some monotonic function of acquired learning (Rescorla and Wagner 1972; Mackintosh 1975; Pearce and Hall 1980; Wagner 1981; Sutton and Barto 1998). To disambiguate learning from performance effects, we employed an associative blocking design (Fig. 3*A*). In a blocking experiment, first an animal is trained such that a cue (cue A) predicts an outcome (pellet). Next, A is presented in compound with a novel cue (cue B) which also leads to the same pellet outcome. If the animal has learned that cue A sufficiently predicts the pellet outcome already, then very little is learned about cue B, that is, learning about cue A blocks subsequent learning about cue B (Kamin 1969). However, if learning about cue A is insufficient, then learning about cue B should not be blocked. We predicted that if OFC inactivation is disrupting learning, then OFC inactivation during initial learning about cue A should disrupt the blocking effect.

First, we demonstrated again that OFC inactivation significantly impairs acquisition in a new cohort of animals using similar parameters to those required for the associative blocking design (inactivation from days 5 to 9 of acquisition with a 10 s visual CS; Supplementary Experiment 2: Supplementary Figure S3 [supplements 1 and 2]). Again, OFC inactivation significantly impaired acquisition, confirming that the effects observed in Figure 2 are not dependent on a specific cue modality or duration.

### Associative Blocking

Next, in a different cohort of animals, we tested whether impaired CS–US acquisition following OFC inactivation disrupted subsequent Pavlovian blocking.

#### Histology and Group Allocation

Cannulae placements are illustrated in Supplementary Figure S3 (supplement 3). One animal failed to consume pellets throughout the experiment and was excluded from testing. One animal from the muscimol group lost its cannula assembly during the infusion period and was excluded from testing. One animal in the muscimol group was euthanized due to severe illness. A further 2 animals were excluded after histological analysis revealed that the cannulae were only unilaterally targeting DLO and LO. Therefore, a total of 6 animals were excluded leaving *N* = 26 (saline *n* = 13, muscimol *n* = 13).

#### PreCS Responding

Baseline levels of responding did not differ between groups during training, and on the final day of infusions (day 10 of stage 1) PreCS response rates (per 10 s) were saline *M* = 0.122, SD = 0.24, muscimol *M* = 0.67, SD = 0.87. These observations were supported by mixed Group x Day ANOVAs on PreCS responding in stage1 suggesting that there were no group differences on days 1–4 prior to infusion (all *F* < 1.69, *P* > 0.21) or on days 5–10 during infusions (significant main effect of Day *F*_(5, 120)_ = 15.21, *P* < 0.001, all remaining *F* < 1.00, *P* > 0.50).

#### Stage 1

During stage 1 of blocking (Fig. 3*B*), all animals were given 10 days of acquisition training to cue A. OFC function was intact during the first 4 days of acquisition, and all animals began to acquire the cue A-outcome relationship (days 1–4: significant main effect of day $F\big(3,72\big)=5.77$, $P=0.001$, but no effect of Group, or Group x Day interaction $F\big(3,72\big)=0.27$, $P=0.850$). All animals then received an additional 6 days of acquisition to cue A (Fig. 3*B*, days 5–10) following either intra-OFC infusions of muscimol or saline. Infusions of muscimol depressed overall responding relative to saline infusions (significant main effect of Group $F\big(1,24\big)=4.25$, $P=0.050$, and day $F\big(\mathrm{5,120}\big)=17.49$, $P<0.001$, but no Group x Day interaction $F\big(\mathrm{5,120}\big)=1.31$, $P=0.263$). Importantly, on the final day (day 10), responding in the muscimol group was significantly lower than the saline group ($t(24)=-2.69$, $P=0.013$).

#### Stage 2

Next, animals were trained such that compounds AB and CD also predicted reward (Fig. 3*C*, Stage 2), importantly OFC function was intact in all animals, that is, no infusions. Responding in both the saline and muscimol groups was initially lower to the novel compound CD than to AB (significant Cue x Day interaction $F\big(2,48\big)=12.12$, $P<0.001$, and main effect of day $F\big(2,48\big)=20.09$, $P<0.001$, but no other main effects or interactions with Group were significant, all remaining effects *F* < 1.91, *P* > 0.160; Cue AB vs. CD: day 12 $t(24)=3.74$, $P=0.001$, day 13 $t(24)=-0.44$, $P=0.663$, day 14 $t(24)=-1.80$, $P=0.085$). However, the pattern of means suggests that responding to compound AB in the muscimol group was similar to the novel compound CD on day 12 (Fig. 3*C*, right—day 12, Muscimol: AB vs. CD $t(24)=1.82$, $P=0.081$), and lower than compound AB in the saline group (Fig. 3*C*, left—day 12; day 12, saline: AB vs. CD $t(24)=3.47$, $P=0.002$). Furthermore, within-session changes over trials on day 12 revealed rapid within-session acquisition to both compounds in both groups, but responding was significantly lower in the muscimol group at the start of the session (see Supplementary Figure S3 [supplement 4]; first 2 trials, significant main effect of Group $F\big(1,24\big)=8.67$, $P=0.007$, and Cue $F\big(1,24\big)=7.61$, $P=0.011$, but no Group x Cue interaction $F\big(1,24\big)=0.19$, $P=0.670$). The lower responding to cue AB in the muscimol group suggests that acquisition to cue A was impaired following infusions in Stage 1 and this impairment persisted (albeit transiently) when test drug free in stage 2. Indeed, the levels of responding to compound AB in the muscimol group at the start of day 12 (see Supplementary Figure S3 [supplement 4]) are similar to levels of responding to the novel compound CD in the saline group. This would suggest that learning about cue A in the muscimol group was impaired in stage 1, and therefore cue A should not effectively block learning to cue B in stage 2.

#### Test

At test both groups showed significant blocking of learning to cue B relative to the control cue D (Fig. 3*D*; Significant main effect of Cue $F\big(1,24\big)=7.29$, $P=0.013$, but no main effect of Group $F\big(1,24\big)=0.54$, $P=0.471$, or Group x Cue interaction $F\big(1,24\big)=0.04$, $P=0.843$). This suggests that inactivation of the OFC significantly reduced behavioral performance but not learning to cue A in Stage 1, and this impairment transiently affected compound AB on day 12 in the absence of OFC inactivation. Therefore, the impairments observed in our earlier findings (Fig. 2*A*, post infusion) are unlikely to be due to impairments in learning. In addition to this, we rule out the possibility that the two groups used different attentional solutions to achieve a similar blocking result (see Supplementary Figure S3 [supplement 5]).

## Discussion

The present studies tested the hypothesis that the rodent lateral OFC is not necessary for Pavlovian acquisition in simple single CS–US procedure. Here, we show that OFC lesions and inactivation significantly affects Pavlovian acquisition. Furthermore, we found a dissociation between pre- and post-training OFC manipulations on Pavlovian acquisition such that pretraining OFC lesions enhance, whereas post-training lesions and inactivation impairs acquisition behavior. Given the absence of these effects in the extant literature, it is notable that these effects were robust and were replicated multiple times. Next, using an associative blocking design, we tested whether impaired behavior following post-training OFC inactivation reflects a disruption of learning or behavioral control. OFC inactivation did not disrupt the underlying learning about the predictive CS–US relationship as assayed by blocking, and instead disrupted the appropriate control of anticipatory behavior to the CS.

### Lateral OFC is Necessary for Simple Pavlovian Acquisition

The significant role of the OFC in Pavlovian acquisition in the present studies is surprising since OFC lesions and inactivation have consistently been reported to have no effect on acquisition in rats (Gallagher et al. 1999; Schoenbaum et al. 2002; Stalnaker et al. 2007; e.g., Burke et al. 2008), unless there are complex cue- or outcome-specific task demands (e.g., Ramirez and Savage 2007). However, in tasks involving simple single Pavlovian CS–US procedures and pretraining OFC lesions, performance often does not reach asymptote (e.g., after 9 days, Gallagher et al. 1999) before proceeding to a new stage of the experiment. In Experiment 1, we did not observe any significant effects of OFC lesions on acquisition until around 15–21 days of acquisition. However, after extended training Schoenbaum et al. (2003) have reported significant effects of OFC lesions on acquisition in a simple cue-outcome go–nogo task when looking at response latencies, but not on trials-to-criterion. Therefore, the effects of pretraining lesions may not have been observed previously due to task specific parameters such as the length of training and the sensitivity of the response measures.

Pretraining OFC lesions have been shown to disrupt Pavlovian acquisition in sign-tracking procedures in which lever insertion is used as the CS (Chudasama and Robbins 2003). Focal lateral OFC lesions also significantly impair sign-tracking behavior (i.e., engaging with the lever cue), and bias behavior towards goal-tacking (i.e., approaching the magazine) (Panayi and Killcross 2018). The present findings that pretraining OFC lesions enhanced behavior focused towards the magazine is consistent with a deficit in sign-tracking and a bias towards goal-tracking.

In contrast to pretraining lesions, post-training OFC inactivation/lesions normally coincide with changes in experimental phase and continued acquisition is not assessed. In tasks in which OFC inactivation coincides with a change in experimental stage, the effects of OFC inactivation are consistent with an impairment in subsequent acquisition (2009). For example, Burke et al. (2009) found that post-training OFC inactivation impaired acquisition to a Pavlovian CS in reversal task. Similarly, Takahashi et al. (2009) found that OFC inactivation during a Pavlovian overexpectation task disrupted new learning. Therefore, the robust effect of impaired acquisition following post-training OFC inactivation that we report is consistent with impaired subsequent acquisition in tasks with more complex manipulations.

### Lateral OFC is not Necessary for Learning the Predictive CS–US Relationship

Post-training OFC inactivation significantly impaired acquisition behavior (Experiment 2), and this disruption was more profound when inactivation occurred earlier in training and more likely to persist after OFC function returned (see Supplementary Experiment 2). This seems to suggest that learning about the CS–US relationship was disrupted. The idea that the OFC could be involved in learning is also consistent with a role for the OFC in the representation of expected values (Burke et al. 2008; Schoenbaum et al. 2011; Stalnaker et al. 2018), which influence midbrain dopaminergic prediction errors (Takahashi et al. 2009, 2011), known to be necessary for Pavlovian learning (Steinberg et al. 2013; Sharpe et al. 2017).

Unexpectedly, the impaired acquisition we observed following post-training OFC disruption did not disrupt the ability of the CS to block learning about a novel cue (Experiment 3, Fig. 3), despite significantly impaired performance postinactivation (see Supplementary Figure S3 [supplement 4]; muscimol AB is as low as saline CD which does not show evidence of blocking). This is surprising given that in some Pavlovian learning contexts, levels of behavioral expression can dictate the extent to which learning occurs (Delamater 2004). This finding highlights the importance of using multiple tests of learning (Rescorla 2002a, 2002b) to assess disrupted acquisition effects.

Intact blocking despite impaired acquisition behavior suggests that OFC inactivation did not disrupt the underlying learning about the associative strength of the CS–US relationship. Associative blocking is often used to assess the role of prediction-error based learning (e.g., Steinberg et al. 2013; Sharpe et al. 2017), suggesting that the OFC is not necessary for this aspect of Pavlovian learning. This distinction suggests that the learned value of a Pavlovian CS–US association might be independent of the current expected or subjective value of expected reward. Informally, learning whether an outcome will be delivered might reasonably be separate from learning the subjective value or identity of that outcome (Delamater 2007; Delamater and Oakeshott 2007; McDannald et al. 2011; Zhou et al. 2019). Indeed, the present findings are consistent with reports that pretraining OFC lesions do not disrupt the blocking effect (McDannald et al. 2011), and neural activity in the lateral OFC in blocking procedures appears to predominantly track sensory specific features of the US (McDannald et al. 2014; Lopatina et al. 2015).

### Pre- vs. Post-Training Effects

The dissociable and opposite effects of pre- and post-training OFC lesions/inactivation on acquisition were surprising and rule out a simple account of OFC dysfunction in terms of prediction-error based learning impairments (see Supplementary Figure S2 [supplement 1]). One possibility is that pretraining lesions result in compensatory function such that learning is supported by other neural systems. In contrast, post-training lesions and inactivation disrupts learning/behavior that has been acquired in an OFC dependent manner. This argument has been proposed when only pretraining OFC lesions (Boulougouris et al. 2007; Boulougouris and Robbins 2009), or only post-training OFC lesions disrupt behavior (Ostlund and Balleine 2007; Balleine et al. 2011). We will also consider two alternative accounts of pre- versus post-training OFC lesion differences based on theoretical accounts of OFC function, sensory-specific outcome expectancy and latent state theories. Note that these theories do not predict an effect of OFC lesions on simple Pavlovian acquisition a priori, and therefore require additional assumptions to account for the present data.

From an associative learning framework, even putatively “simple” single cue-outcome Pavlovian learning can involve a number of different psychological/behavioral processes (Konorski 1967; Mackintosh 1974; Holland 1977; Dickinson 1980; Rescorla 1988; Hall 2002). Take for example a 10 s light cue that reliably predicts the delivery of a pellet reward. A rat can learn that the cue predicts the sensory-specific properties of the outcome (e.g., taste, texture, sweetness, color, size, location etc.), or the general motivational value of that reward, or simply develop a stimulus–response habit to approach the reward location when the cue is presented. Indeed, there is experimental evidence for these multiple aspects of learning occurring during Pavlovian conditioning (for review, see Delamater and Oakeshott 2007). It is possible that pretraining OFC lesions disrupt the balance of these different aspects of Pavlovian learning and behavior (Burke et al. 2007; Delamater 2007).

If the OFC is necessary for the representation of the sensory specific properties of expected outcomes, then OFC lesions might allow a stimulus–response habit system to dominate behavioral control. Following pretraining lesions, this may lead to an unconstrained habit learning system (Dickinson 1985; Coutureau and Killcross 2003; Killcross and Coutureau 2003; Dolan and Dayan 2013) that is not necessarily bounded by the current value of the outcome, and overly sensitive to current general motivational states (e.g., overall hunger levels; Figure 1B) of the organism. This is consistent with evidence that a stimulus–response habit like system develops in Pavlovian conditioning paradigms (Hall 2002; Killcross and Blundell 2002; Parkinson et al. 2005), and is likely to interact and compete with stimulus-outcome learning systems for behavioral control, similar to the interaction found between instrumental habit and goal-directed systems (Coutureau and Killcross 2003; Killcross and Coutureau 2003; Yin et al. 2005, 2006; Balleine and Killcross 2006; Lee et al. 2014; Kim et al. 2019), except that in Pavlovian conditioning the cue-outcome system often dominates in control animals (Holland et al. 2008). However, once initial learning occurs with an intact OFC, the encoding of the identity of the expected outcome is likely to have occurred (e.g., Delamater and Holland 2008). Subsequently, a post-training lesion or inactivation of the OFC is likely to affect the subsequent updating of this information. Therefore, one possible account is that the impaired acquisition behavior we observed following post-training inactivation reflects an inability to update the current motivational value of the specific outcome that is expected.

The latent state representation account of the OFC might also be able to account for the differences observed dissociation between pre- and post-training OFC lesions on acquisition. Computational models (e.g., Wilson et al. 2014) often assume, for simplicity, that in a simple single cue-outcome procedure, the cue state (e.g., “light on”) is stable throughout acquisition. Given that the same cue is presented, and it always leads to the pellet outcome, this stable representation is a reasonable assumption. However, it is also likely that early in acquisition, this state representation is not yet stable in healthy control animals (Niv 2019). How can the animal be certain that the light cue, the testing chamber context, or the reward pellet that they see on each trial is identical to the trials they have already experienced within the session, and from previous days? The subjective experience of these states and their physical features is very likely to be different within- and between-sessions, for example, the ambient noises, odors, temperature of the context, the location and intensity of the light cue based on where the rat happens to be located when it turns on, and the gradual onset of sensory specific satiety to the pellet, etc. Informally, how does the rat know that this light is the same light that they saw at the start of the session, or the day before? The perception and recognition of these states is therefore subject to differences in variables such as generalization, confidence, and certainty.

Paradoxically, in a simple and stable cue-outcome training procedure, pretraining OFC lesions may result in an accurate, but inflexible, representation of these simple task states quite rapidly. In this stable and simple training context, this could lead to enhanced Pavlovian acquisition. However, in a task with multiple or uncertain cue-outcome contingencies pretraining OFC lesions might impair acquisition (Walton et al. 2010; Stolyarova and Izquierdo 2017). However, post-training inactivation of the OFC would disrupt the ability to update already established state representations at whatever stage of certainty/stability that they have currently achieved. In the stable single cue-outcome learning situation employed in the present studies, this would result in disruption of further acquisition. Again, in a task with interference from multiple cue-outcome relationships, post-training lesions might improve performance.

## Conclusion

Here, we show that the rodent lateral OFC is involved in Pavlovian acquisition learning process in an experience dependent manner. Once initial learning has taken place, the lateral OFC appears to be necessary for updating the current value of Pavlovian behaviors driven by expected outcome value. These findings raise two important issues. First, they demonstrate the importance of not interpreting a null effect of lesions on acquisition behavior as evidence that the OFC is not involved in acquisition learning. Instead, the underlying deficit in acquisition either is not being expressed or is not relevant to behavioral performance in the task yet. Second, these findings demonstrate that even within a putatively “simple” behavioral task, there are many potential underlying psychological processes that can contribute to performance and change over time. This is consistent with growing suggestions that the competition and interaction between underlying learning systems (Lee et al. 2014; e.g., Kool et al. 2018; Kim et al. 2019) is important and needs further study (Collins and Cockburn 2020).

Recently, we demonstrated functional heterogeneity within the lateral OFC between anterior and posterior subregions (Panayi and Killcross 2018). While the present experiments did not explicitly target and compare anterior and posterior subregions, it is notable that present lesion and cannula placements targeted predominantly anterior lateral OFC. Therefore, one possible account of the surprising role of lateral OFC in simple Pavlovian acquisition is that prior research has often focused on the posterior lateral OFC or the structure as a whole (Gallagher et al. 1999; Ostlund and Balleine 2007; Izquierdo 2017). However, further studies systematically comparing anterior and posterior subregions within lateral OFC are still needed.

While the OFC has often been found not to be necessary for initial acquisition learning, recently, there have been reports that simple Pavlovian acquisition is significantly impaired rather than enhanced following optogenetic inhibition of OFC function in head fixed mice (Namboodiri et al. 2019; Wang et al. 2020), in a manner that does not depend on VTA prediction error signaling. In contrast to our results, these studies target more ventral and medial OFC, which is likely to be an important anatomical distinction given the emerging evidence of functional heterogeneity within the OFC (Sharpe et al. 2015; Barreiros et al. 2020; Bradfield and Hart 2020; Barreiros et al. under review). Indeed, there appears to be dissociable but complementary roles of the medial and lateral OFC such that lateral OFC lesions disrupt Pavlovian whereas medial OFC lesions disrupt instrumental behavioral control (Ostlund and Balleine 2007; McDannald et al. 2011; Bradfield et al. 2015, 2018; Gardner et al. 2017, 2018; Panayi and Killcross 2018). This suggests that the OFC, as a whole, is engaged in the learning and flexible updating of value-based behaviors, but within the orbital subregions this process appears to be remarkably specialized for distinct types of behavior and learning.

## Supplementary Material

Panayi_Killcross_CCC_OFCinPavlovianAcquisition_Supplementaries_FinalFullSubmission_tgab010

## Notes

We gratefully acknowledge Fred Westbrook, Nathan Holmes, David Bannerman, Mark Walton, Mehdi Khamassi, and Geoffrey Schoenbaum for their invaluable feedback. *Conflict of Interest*: The authors declare no competing interests.
