# Supplementary material for: The Role of the Rodent Lateral Orbitofrontal Cortex in Simple Pavlovian Cue-Outcome Learning Depends on Training Experience
Source: Cereb Cortex Commun. 2021 Feb 9;2(1):tgab010. doi: 10.1093/texcom/tgab010 (PMC8152875; doi:10.1093/texcom/tgab010)
Supplement: Panayi_Killcross_CCC_OFCinPavlovianAcquisition_Supplementaries_FinalFullSubmission_tgab010 [file panayi_killcross_ccc_ofcinpavlovianacquisition_supplementaries_finalfullsubmission_tgab010.docx]

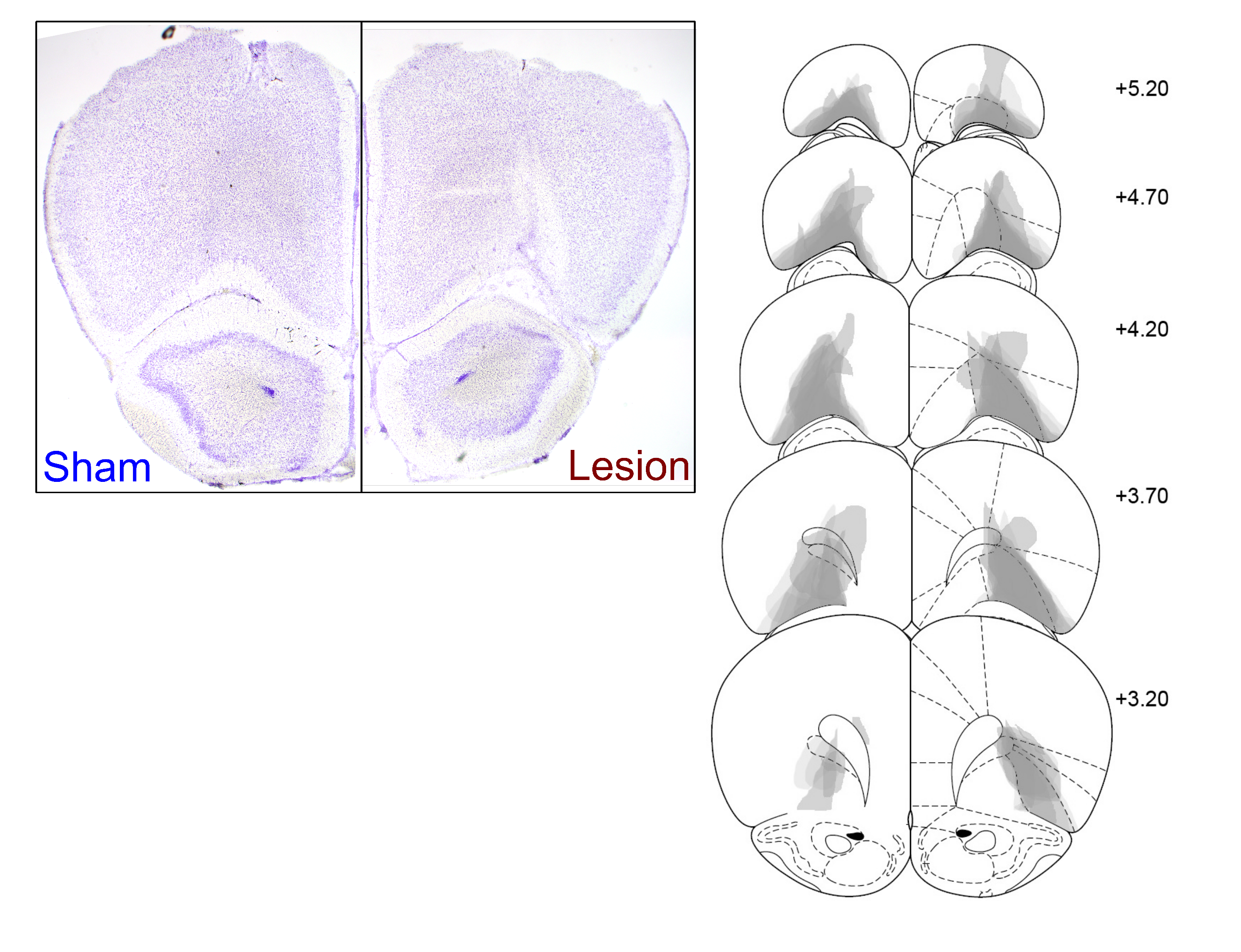


**Figure 1-figure supplement 1.** Photomicrograph of representative OFC lesion damage (Left) in the sham and lesion groups. Coronal slice located approximately +4.20 mm anterior to bregma. Semi-transparent grey patches (Right) represent lesion damage in each subject, and darker areas represent overlapping damage across multiple subjects. Coronal sections are identified in mm relative to bregma (Paxinos and Watson, 1997).

**
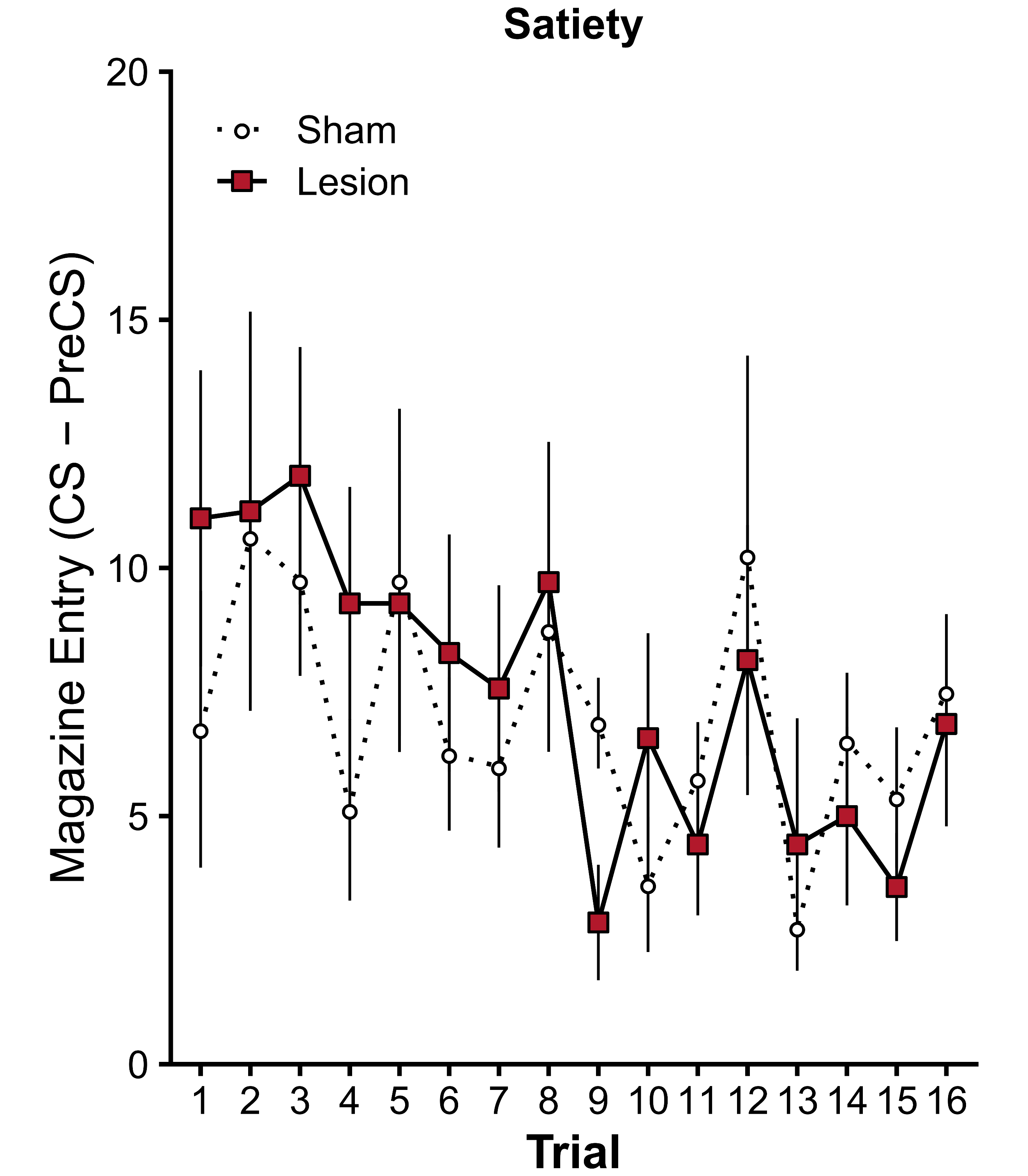
**

**Figure 1-figure supplement 2.** Responding on each trial within-session is presented following a general satiety manipulation (session average presented in Figure 1B). Conditioned responding was not significantly different between the lesion and sham group, and this is evident on trial 1 prior to contact with the reward ($t(13)=1.04$, $p=.317$). Error bars depict ± SEM.


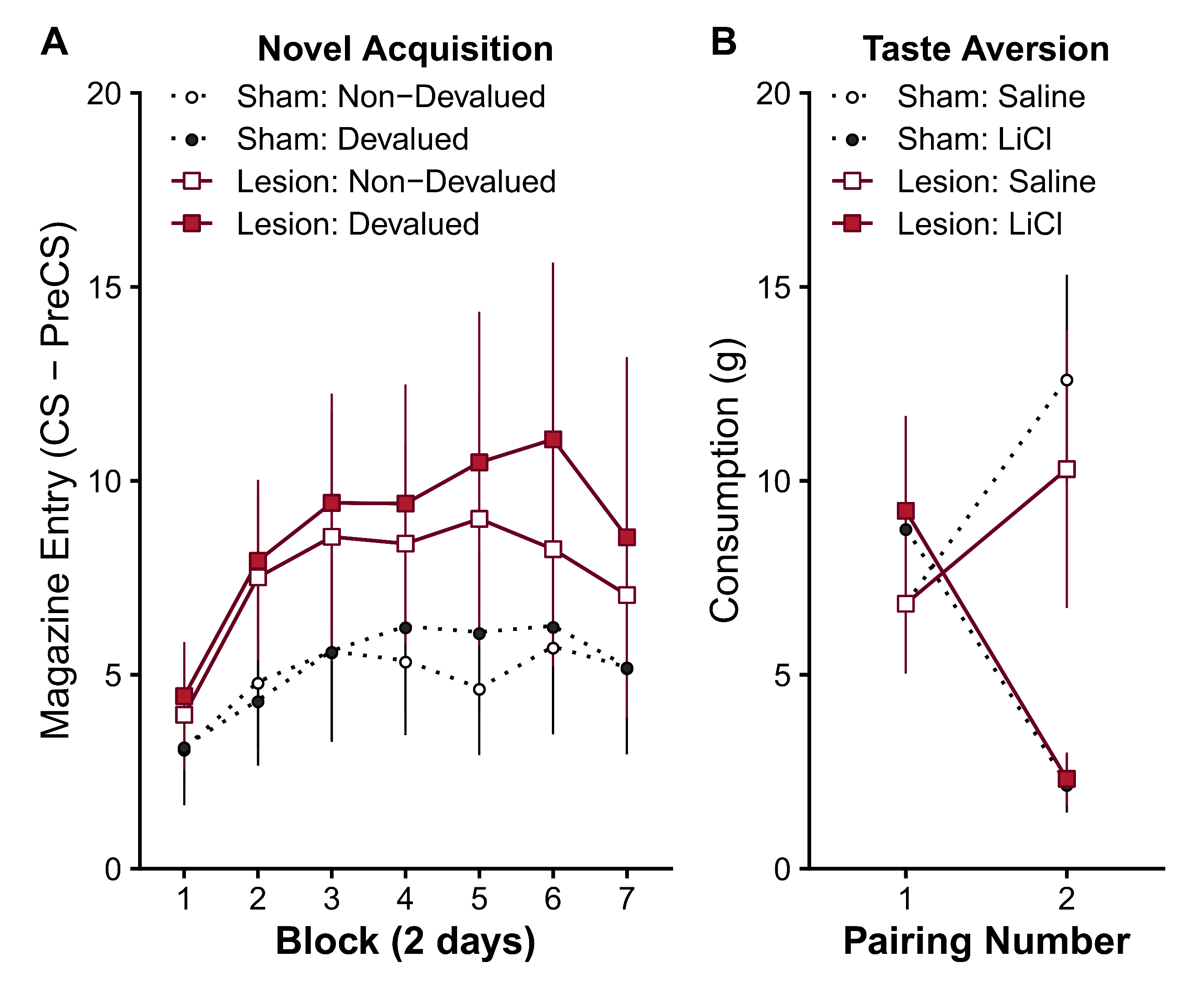


**Figure 1-figure supplement 3.** (**A**) CS-PreCS response levels during the acquisition of two novel and distinct cue-outcome pairings. One outcome was designated for subsequent devaluation and responding is presented separately for the cue that predicts the to-be devalued and non-devalued outcomes. While mean response rates suggested elevated responding in the lesion group, this observation was not supported statistically (main effect of Block $F(6,66)=3.96$, $p=.002$, all remaining effects *F* < 0.98, *p* > .343). It is likely that the elevated responding in the lesion group did not reach significance due to the reduced number of subjects in the lesion group. (**B**) Average consumption (g) of the outcome followed immediately followed by an injection of saline or LiCl to establish a selective taste aversion. All animals acquired a significant taste aversion to the outcome paired with post-consumption LiCl injections (significant Injection x Pairing interaction $F(1,11)=49.58$, $p<.001$, all remaining effects *F* < 2.03, *p* > .182), which did not significantly differ between lesion groups. Specifically, consumption of the outcome paired with LiCl significantly decreased between pairing 1 and 2 (LiCl: pairing 1 vs 2 $t(11)=4.26$, $p=.001$), whereas consumption of the outcome paired with saline increased (saline: pairing 1 vs 2 $t(11)=-3.74$, $p=.003$). Error bars depict ± SEM.


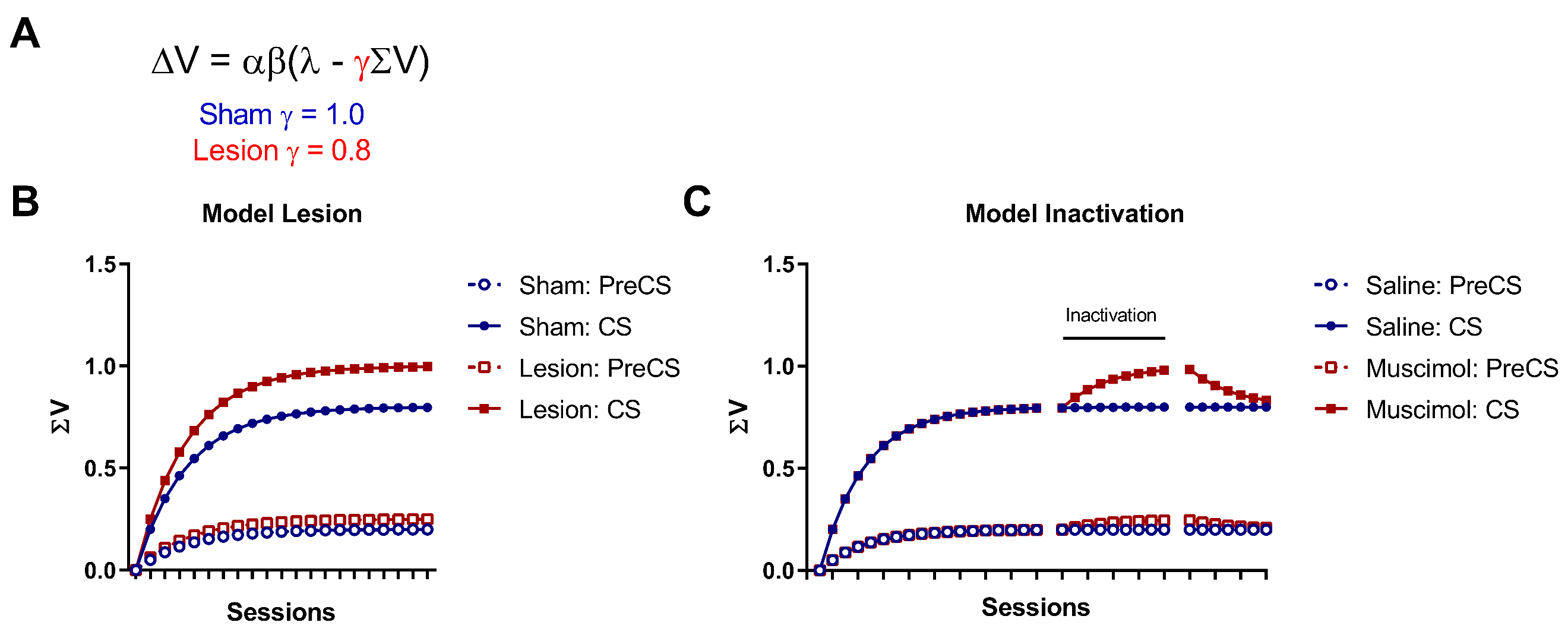


Figure 2-figure supplement 1. Model predictions of LO lesions as a partial loss of outcome expectancy. (A) The model predictions for simple Pavlovian acquisition using a standard Rescorla-Wagner learning rule and following modelled lesion deficits as a partial loss of outcome expectancy information specific to learning. Using the following update rule: $\Delta V= \alpha\beta\left( \lambda-\gamma\Sigma V \right)$, the change in associative strength ($\Delta V$) was calculated on a trial-by-trial basis for the CS and the context (preCS) periods. Parameters used were$\alpha\beta$ = .05 for the context and .2 for the CS, $\lambda$ = 1, and γ = 0.8 for lesions and γ = 1.0 for the control condition. **(B)** Modelled performance following pre-training sham or LO lesions. **(C)** Modelled performance following temporary LO inactivation via muscimol after initial training.

One potential account of the heightened responding observed during simple acquisition following LO lesions is enhanced cue-outcome learning. However, given the hypothesized role of the OFC in the representation of outcome expectancy value (Baxter et al. 2000; Pears et al. 2003; Schoenbaum et al. 2009; Takahashi et al. 2009; Takahashi et al. 2011), it is not immediately clear how this could be the case. The Rescorla-Wagner model of the acquisition of associative value suggests that in the absence of a representation of the expected value of an outcome, no conditioned responses should be expressed. In fact, in most associative learning theories the rules that govern performance are some function of the expected value of the outcome (Rescorla and Wagner 1972; Mackintosh 1975; Pearce and Hall 1980; Sutton and Barto 1998; LePelley 2004; Esber and Haselgrove 2011; Nasser et al. 2017). However, OFC damage may not abolish all outcome expectancy information but instead might degrade some aspect (e.g. sensory specific properties) of the outcome expectancy information made available at the time of calculating the prediction error (e.g. mid brain Dopamine neurons; Takahashi et al. 2011). This would assume that the expected value used in prediction error learning may not necessarily be the same as the full expected value used to govern performance. To model this mathematically, a constant (γ) is used to represent the proportion of the outcome expectancy information that was available for learning (Figure 2-Figure supplement 1A). The strength/value (V) of the learned association changes in the following manner:

$$\Delta V= \alpha\beta\left( \lambda-\gamma\Sigma V \right)$$

Such that $\Delta V$ is the change in associative strength on a given trial; $\alpha\beta$ are learning rate parameters based on the properties of the cue and outcome respectively such that $0\leq\alpha\leq1$ and $0\leq\beta\leq1$; $\lambda$ is the experienced value of the outcome on a given trial; $\Sigma V$ is the sum of the associative strength i.e. the expected value. The constant γ represents the proportion of $\Sigma V$ available for learning such that $0\leq\gamma\leq1$. In a healthy control animal 100% (γ = 1) of the available outcome expectancy information is available to guide learning. Consequently, further learning will stop once $\lambda=\Sigma V$ as the current expected value of the outcome fully accounts for the actual value of the outcome. Following OFC lesions some of this information is lost and, for example, 80% (γ = 0.8) of the available outcome expectancy information is available to guide learning. Therefore, learning will continue beyond the point at which $\lambda=\Sigma V$ and will only stop once $\lambda=\gamma\Sigma V$. Therefore, the asymptote of conditioned behaviour (expressed as some function of $\Sigma V$) will be determined by $\Sigma V=\frac{\lambda}{\gamma}$ , therefore asymptotic conditioned behaviour will be higher following a lesion in this model ($\gamma<1)$ than an intact OFC ($\gamma=1)$.

The following simulation of this model (with the lesion parameter set at γ = 0.8) reveals a pattern of acquisition that is similar at the start of acquisition but diverges considerably towards the end of acquisition. This pattern strongly resembles the findings in Figure 1A with a range of values of γ > 0.6. This provides prima facie evidence of how well the model accounts for the present data; however, the most valid test of the model would be an experimental test of a hypothesis generated from the model. This model predicts that if acquisition initially occurs with a functional LO until asymptote (i.e. $\Sigma V=\lambda$) and no more learning is evident, inactivation of OFC should allow learning to temporarily increase above asymptote because $\lambda<\gamma\Sigma V$. Informally, the inactivation of LO will cause the outcome to no longer be fully predicted by the CS because part of the expectation is now missing. Similarly, if the function of LO is returned, then any new learning will extinguish until $\lambda=\Sigma V$ again. This prediction is modelled in (**C**). It is noteworthy that modelling the effect of LO damage in this way produces identical predictions in a number of other associative learning models (Esber & Haselgrove, 2011; Mackintosh, 1975; Pearce & Hall, 1980; Rescorla & Wagner, 1972) and therefore may also affect other aspects of learning such as attention. Furthermore, even though the hypothesis was generated using the current mathematical model, the prediction and underlying mechanism would be the same for any interpretation of the increased responding in Figure 1A as increased cue-outcome learning. If OFC damage increases learning, then transient inactivation of OFC should temporarily allow learning to increase.

**
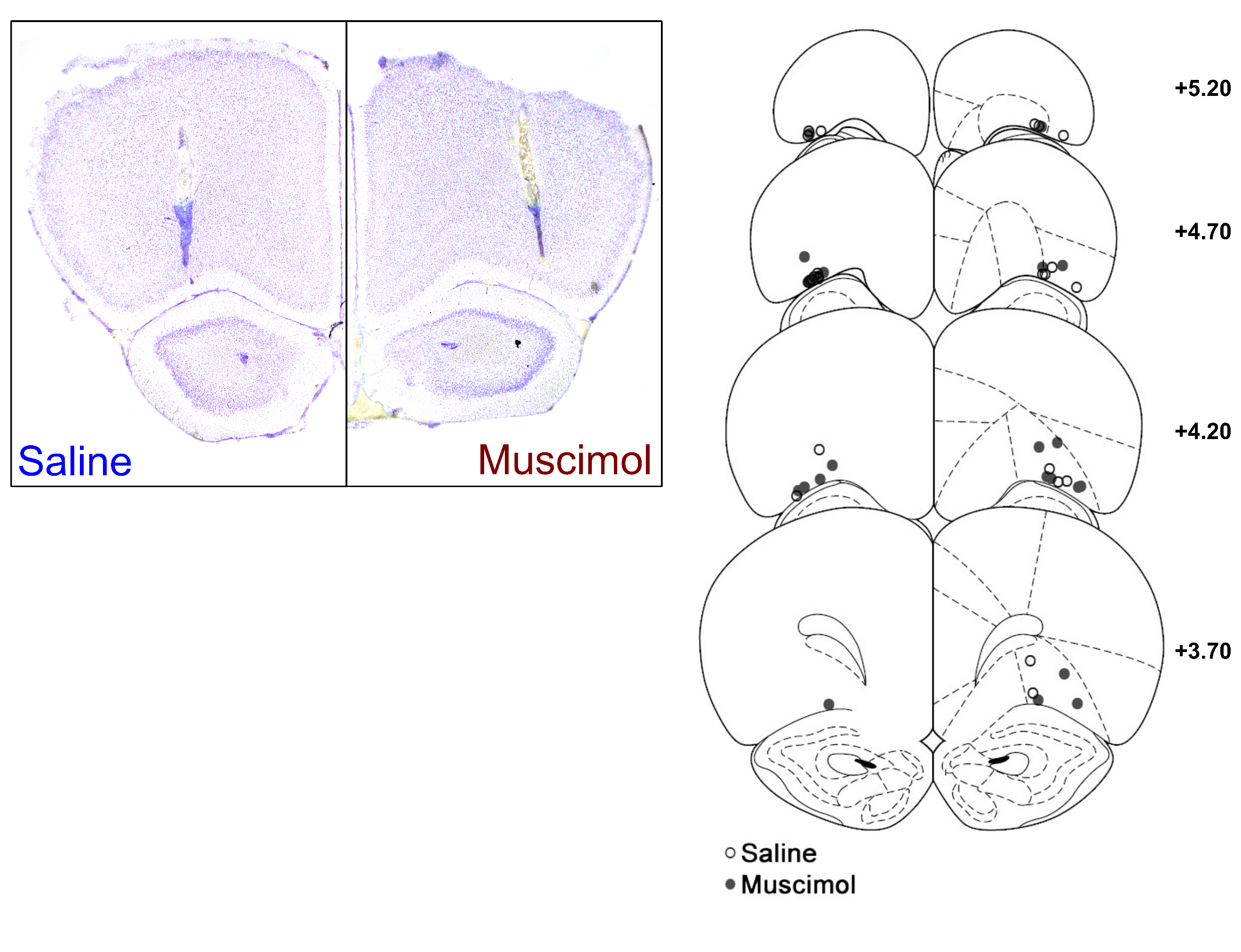
**

**Figure 2-figure supplement 2.** Photomicrograph of representative OFC cannulae placement (Left) in the saline and muscimol infusion groups. Coronal slice located approximately +4.20 mm anterior to bregma. Cannulae tip (Right) location of all subjects in the saline (empty circles) and muscimol (filled circles) groups. Coronal sections are identified in mm relative to bregma (Paxinos and Watson, 1997).

**Supplementary Experiment 1: Post-Training OFC lesions**

**Histology and Group Allocation**

Lesion damage is depicted in Figure 2-figure supplement 3. Lesion extent was judged by a trained observer blind to group allocation. A lesion was retained if there was evidence of significant bilateral damage constrained to LO or DLO. Animals were excluded if there was only unilateral LO/DLO damage, evidence of damage to the dorsal part of the anterior olfactory nucleus ventral to LO/DLO or if there was extensive damage to the white matter of the forceps minor of the corpus callosum. Three lesion animals had only unilateral OFC damage and were excluded from analysis (final *N* = 21; sham *n* = 12, lesion *n* = 9).


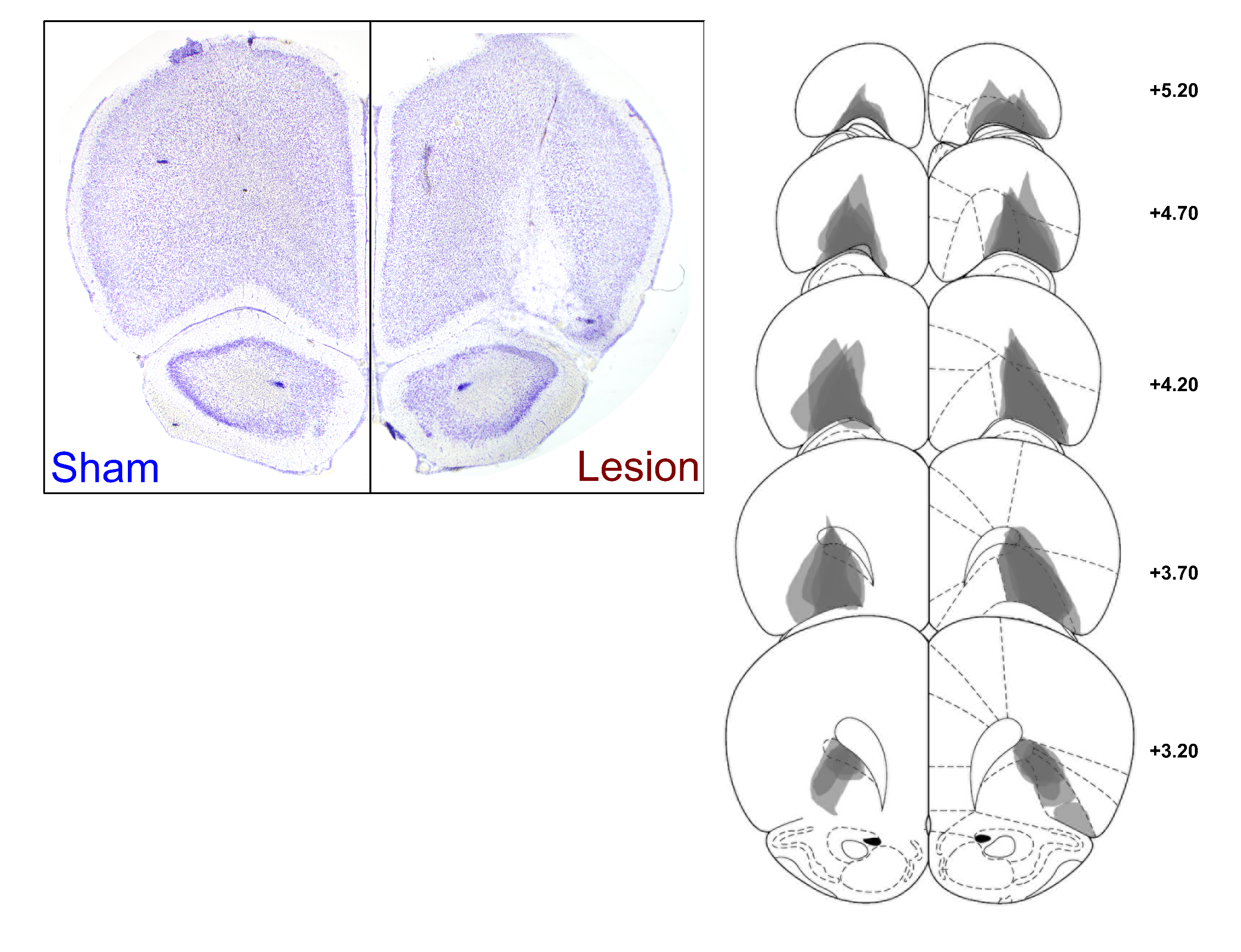


**Figure 2-figure supplement 3.** Supplementary Experiment 1: Photomicrograph of representative OFC lesion damage (Left) in the sham and lesion groups following post-training lesions (behavioural data depicted in Figure 2-figure supplement 4). Coronal slice located approximately +4.20 mm anterior to bregma. Semi-transparent grey patches (Right) represent lesion damage in each subject, and darker areas represent overlapping damage across multiple subjects. Coronal sections are identified in mm relative to bregma (Paxinos and Watson, 1997).

**PreCS Responding**

PreCS levels of responding did not differ between groups across days of training, and on the final block of 3 days (post-operative) response rates (15s) were sham *M* = 2.55, *SD* = 2.03, lesion *M* = 2.74, *SD* = 0.94. A mixed Group x DayBlock (6 blocks of 3 days) ANOVA on preCS responding supported this observation with only a significant main effect of DayBlock (*F*_(5, 95)_ = 11.52, *p* < .001, effect of Group and Group x DayBlock interaction *F* < 1.00, *p* > .81).

**Acquisition**

We trained a new cohort of animals on this simple Pavlovian cue-outcome task for 9 days, and then performed post-training excitotoxic or sham OFC lesions before continuing with acquisition (lesion extent depicted in Figure 2-figure supplement 3). Prior to surgery, animals acquired responding to the cue (Pre-Surgery; significant main effect of Block $F(2,38)=61.98$, $p<.001$, but no main effect of Group $F(1,19)=0.49$, $p=.492$, or Group x Block interaction $F(2,38)=0.31$, $p=.738$). After surgery, the sham group continued to acquire responding, but the lesion group did not (Post-Surgery; significant Group x Block interaction $F(2,38)=6.25$, $p=.005$ , but no main effect of Group $F(1,19)=2.21$, $p=.154$, or Day $F(2,38)=0.66$, $p=.525$). Responding in the sham control group was significantly higher than the lesion group in the final block of 3 days (Block 4 $t(19)=-0.25$, $p=.806$, Block 5 $t(19)=-0.80$, $p=.434$, Block 6 $t(19)=-2.65$, $p=.016$). Furthermore, further acquisition post-surgery was completely abolished in the lesion group (Lesion: no linear trend over Blocks 4-6 $t(19)=-1.42$, $p=.172$), but continued in the sham control group (Sham: significant positive linear trend over Blocks 4-6 $t(19)=2.93$, $p=.009$. Therefore, both post-training lesions and inactivation of OFC function disrupted Pavlovian acquisition.

To facilitate comparisons between experiments, CS-PreCS response rates on Block 6 in the present experiment were Sham: M = 9.61, SD = 3.88, Lesion: M = 7.18, SD = 1.74. The terminal levels of responding in the sham group are similar to those of the saline group in Figure 2, and the sham group in Figure 1A which used identical session parameters. This suggests that the present findings are also unlikely to be due to abnormally elevated levels of responding in the control groups in any one of these experiments.


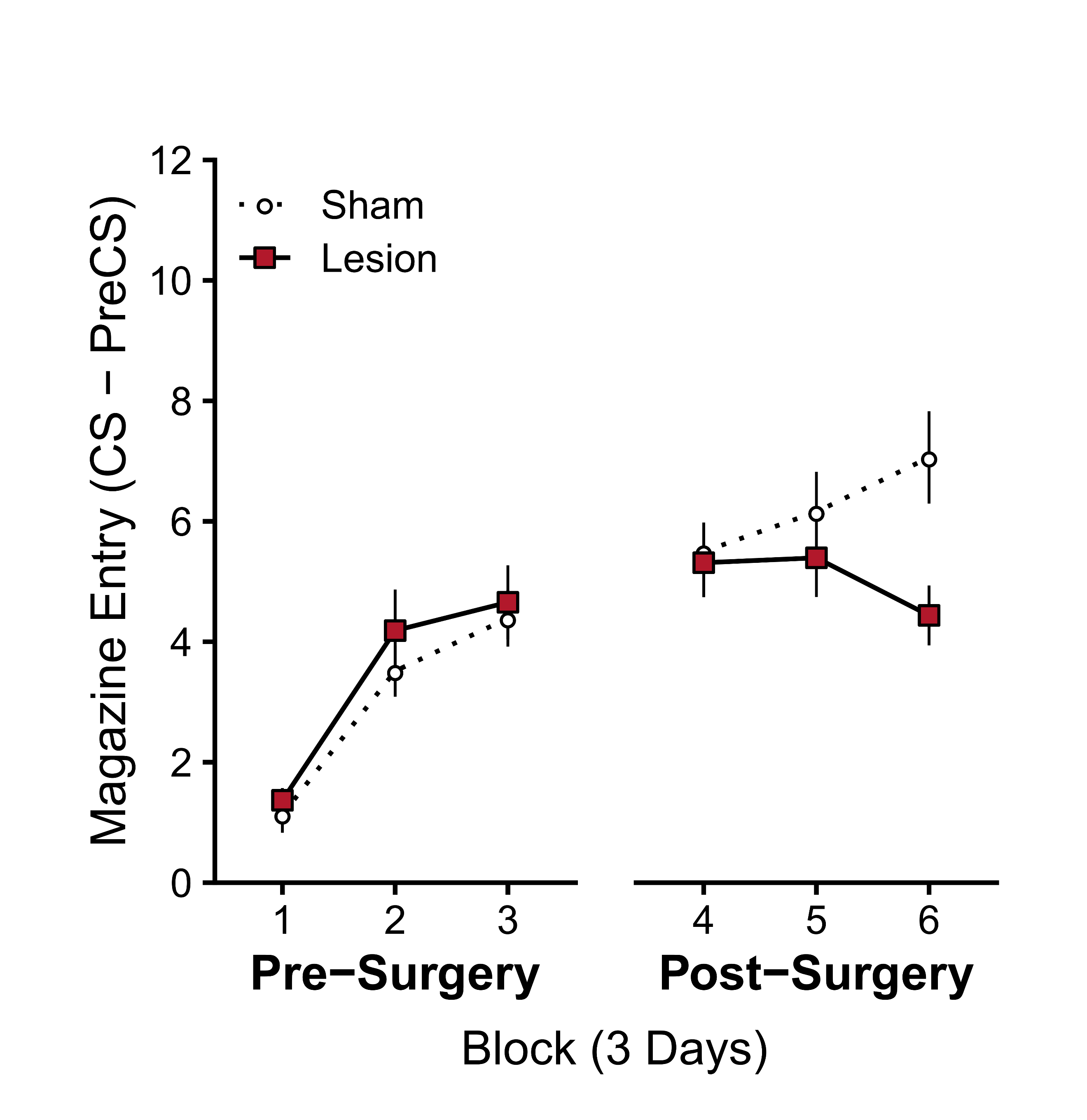


**Figure 2-figure supplement 4.** Supplementary Experiment 1: Post-training OFC lesions suppress Pavlovian acquisition behaviour. Rates of discriminative responding (CS-PreCS) during initial acquisition (Pre-Surgery, Blocks 1-3), and following OFC or sham lesions (Post-Surgery, Blocks 4-6). Data summarized in blocks of 3 days. Histological characterization of lesion extent depicted in Figure 2-figure supplement 3. Error bars depict ± SEM.

**Supplementary Experiment 2: OFC inactivation early in acquisition**

**Histology and exclusions**

Cannulae placements are illustrated in Figure 3-figure supplement 1. One rat in the Muscimol condition had a blocked guide cannulae and was excluded from experimental analysis. Final numbers N = 15 (Muscimol n = 7, Saline n = 8).

**
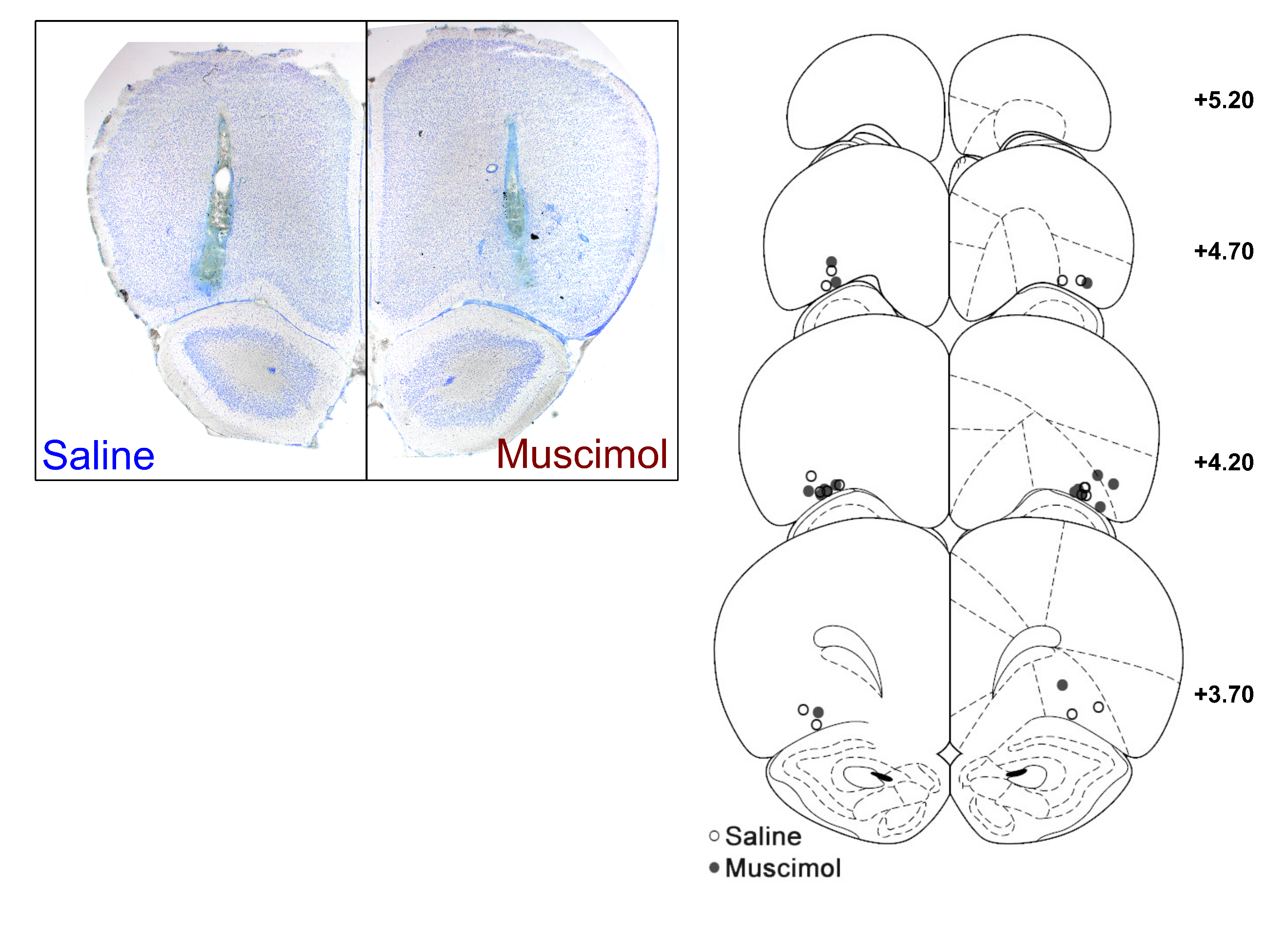
**

**Figure 3-figure supplement 1.** Supplementary Experiment 2: Photomicrograph of representative OFC cannulae placement (Left) in the saline and muscimol infusion groups (associated behavioural data presented in Figure 3-figure supplement 1). Coronal slice located approximately +4.20 mm anterior to bregma. Cannulae tip (Right) location of all subjects in the saline (empty circles) and muscimol (filled circles) groups. Coronal sections are identified in mm relative to bregma (Paxinos and Watson, 1997).

**PreCS Rates**

PreCS responding did not differ between infusion groups across the 10 days of Pavlovian conditioning (Group *F*_1,13_ = 2.72, *p* = .12; Day *F*_9,117_ = 1.49, *p* = .16; Group x Day *F*_9,117_ = 2.72, *p* = .25).

**Acquisition**

Prior to drug infusions, all animals acquired responding to the cue (Figure 2C, Days 1-4; Significant main effect of Day $F(3,39)=9.42$, $p<.001$, but no main effect of Group , or Group x Day interaction $F(3,39)=0.30$, $p=.826$). However, OFC inactivation during the next 5 days of conditioning significantly impaired acquisition in the muscimol group (Days 5-9; Significant main effect of Group $F(1,13)=12.57$, $p=.004$, Day $F(4,52)=7.72$, $p<.001$, and Group x Day interaction $F(4,52)=3.05$, $p=.025$). Responding in the muscimol group was significantly lower than the saline group on days 7-9 (Muscimol vs Saline: Day 5 $t(13)=-1.85$, $p=.087$, Day 6 $t(13)=-2.10$, $p=.056$, Day 7 $t(13)=-3.77$, $p=.002$, Day 8 $t(13)=-4.17$, $p=.001$, Day 9 $t(13)=-3.80$, $p=.002$). Again, this deficit was characterised by significant acquisition over days in the saline group that was abolished in the muscimol group (positive linear trend over days 5-9; Saline $t(13)=6.59$, $p<.001$, Muscimol $t(13)=1.45$, $p=.171$). Finally, this reduction in responding persisted on day 10 when all rats were tested without infusion (Day 10; $t(12.14)=3.88$, $p=.002$). In contrast to OFC inactivation later in acquisition (Figure 2), disrupting OFC activity early in learning suppressed performance which persisted when the OFC was active again. These findings suggest that OFC inactivation early in training disrupted acquisition learning rather than just behavioural performance.


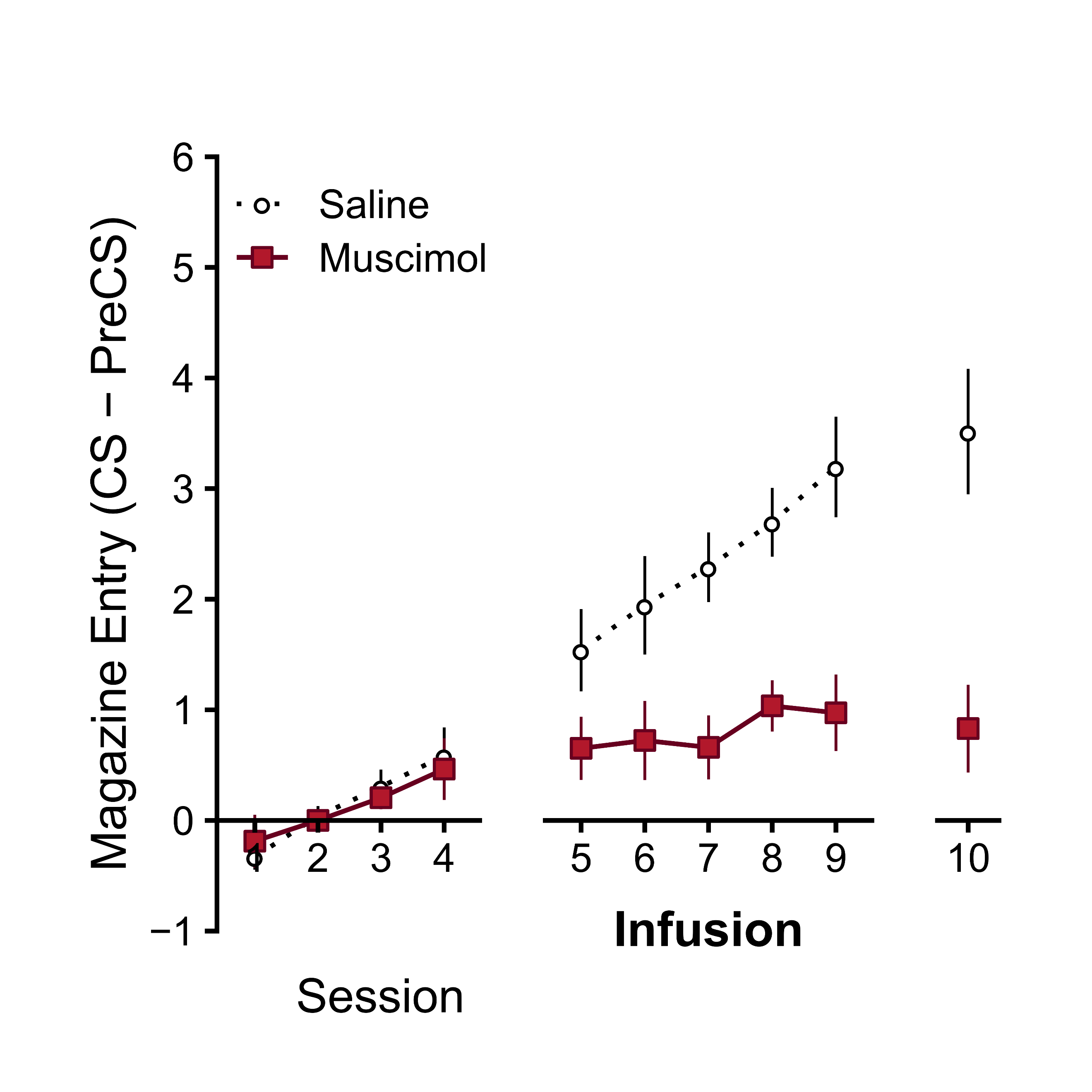


**Figure 3-figure supplement 2.** Supplementary Experiment 2: Post-training OFC inactivation early in training suppresses Pavlovian acquisition behaviour, and this suppression persists in the absence of OFC inactivation. Rates of discriminative responding (CS-PreCS) during initial acquisition (sessions 1-4), following intra-OFC infusion of muscimol or saline (sessions 5-9), and without infusion (session 10). Cannulae placements depicted in Figure 3-figure supplement 1. Note that the task presented in Figure 1 and Figure 2 employed a 15s auditory CS, whereas a 10s visual CS was used in the present figure and during the associative blocking experiment presented in Figure 3. Stimulus modality, salience, and duration differences account for the different levels of conditioned responding between these experiments. Error bars depict ± SEM.


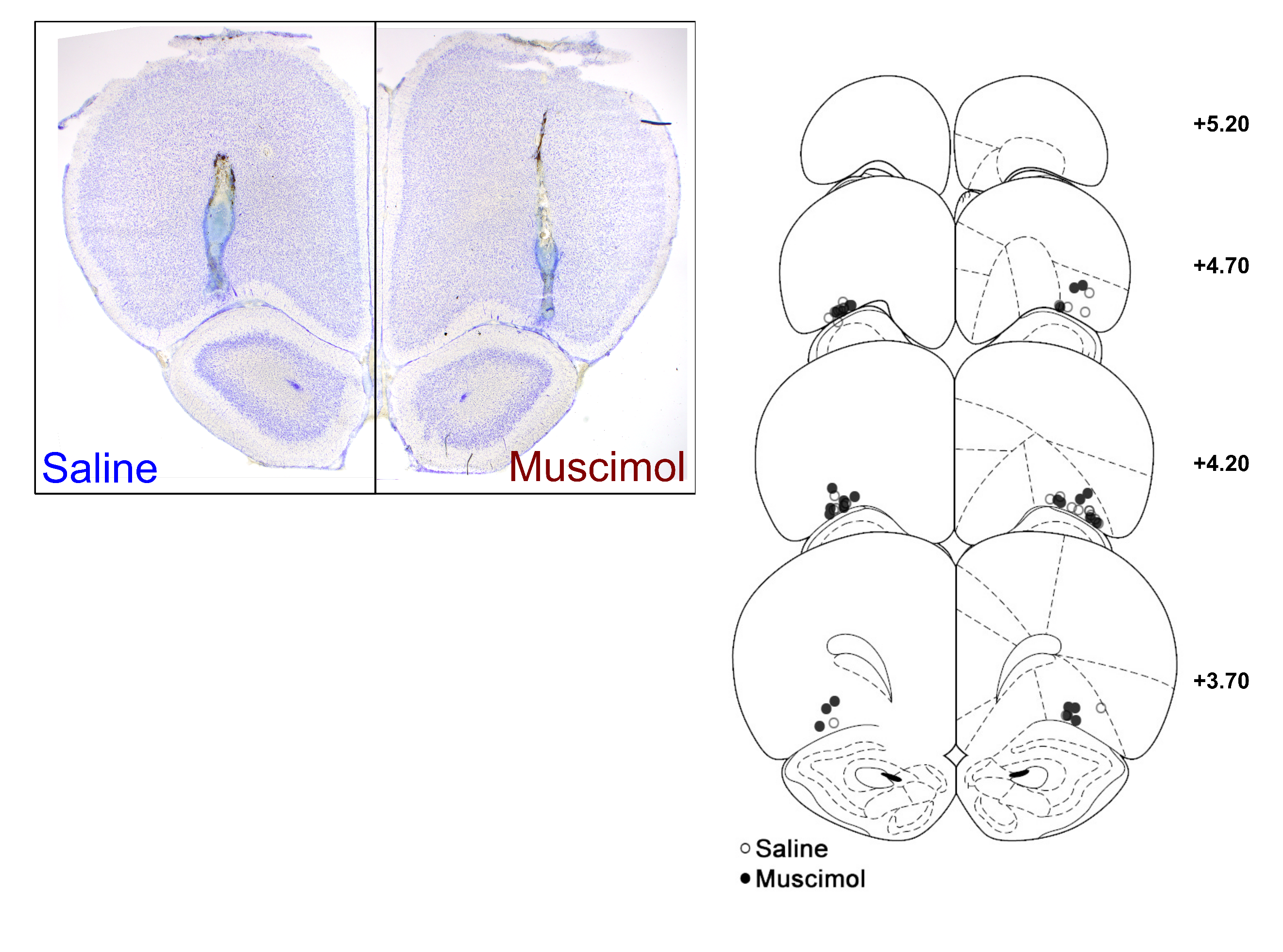


**Figure 3-figure supplement 3.** Photomicrograph of representative OFC cannulae placement (Left) in the saline and muscimol infusion groups (associated behavioural data in Figure 3). Coronal slice located approximately +4.20 mm anterior to bregma. Cannulae tip (Right) location of all subjects in the saline (empty circles) and muscimol (filled circles) groups. Coronal sections are identified in mm relative to bregma (Paxinos and Watson, 1997).

**
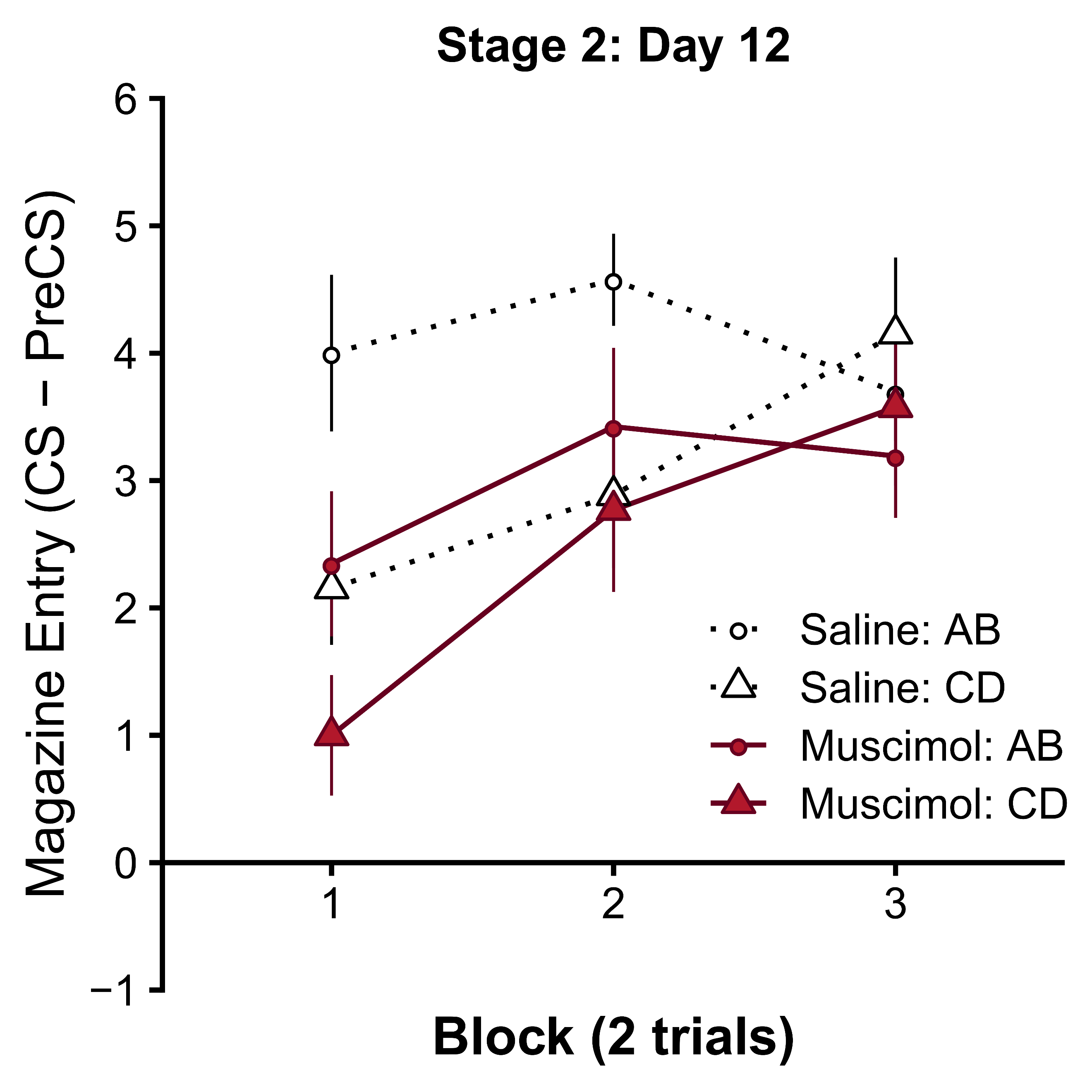
**

**Figure 3-figure supplement 4.** Within-session responding on Day 12, the first session of Stage 2 blocking (session average shown in Figure 3C). Rates of discriminative responding (CS-PreCS) presented in blocks of 2 trials. The effect of muscimol inactivation in Stage 1 is evident at the start of the session i.e. a significant reduction in responding relative to the saline group. First 2 trials, significant main effect of Group $F(1,24)=8.67$, $p=.007$, and Cue $F(1,24)=7.61$, $p=.011$, but no Group x Cue interaction $F(1,24)=0.19$, $p=.670$). Error bars depict ± SEM.

**
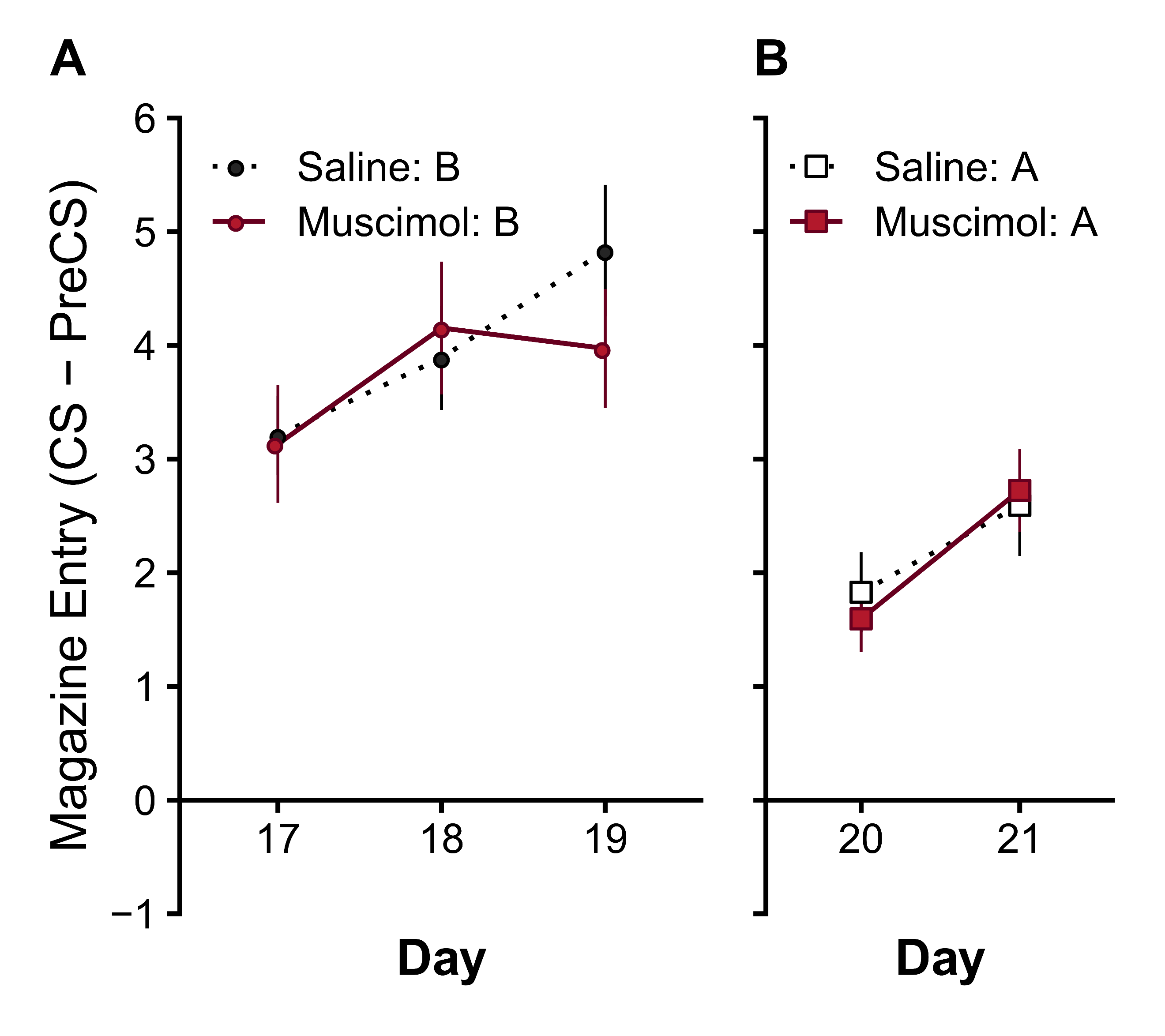
**

**Figure 3-figure supplement 5.** Reacquisition to cues A and B provide a test of whether the successful blocking effect observed in the saline and muscimol groups was the result of different underlying attentional strategies. Down-regulation of attention to a cue can result in retardation of subsequent acquisition (Mackintosh 1975; Pearce and Hall 1980; Papini and Bitterman 1993). There were no differences in the rates of learning to the blocked cue B or to the blocking cue A. (**A**) The rate of re-acquisition to cue B (day 17-19) and (**B**) cue A (day 20-21) did not differ between groups (Cue B: Significant main effect of Day $F(2,44)=9.54$, $p<.001$, but no main effect of Group $F(1,22)=0.12$, $p=.727$, or Group x Day interaction $F(2,44)=1.99$, $p=.148$. Cue A: Significant main effect of Day $F(1,22)=54.73$, $p<.001$, but no main effect of Group $F(1,22)=0.01$, $p=.919$, or Group x Day interaction $F(1,22)=2.02$, $p=.169$). Note: Due to experimenter error one animal in each group was tested with the wrong counterbalancing and excluded from the analysis of re-acquisition data (remaining N = 24, saline n = 12, muscimol n = 12). Error bars depict ± SEM.

**References**

Baxter MG, Parker A, Lindner CC, Izquierdo AD, Murray EA. 2000. Control of response selection by reinforcer value requires interaction of amygdala and orbital prefrontal cortex. J Neurosci. 20(11):4311–4319.

Esber GR, Haselgrove M. 2011. Reconciling the influence of predictiveness and uncertainty on stimulus salience: a model of attention in associative learning. Proc R Soc B-Biological Sci. 278(1718):2553–2561.

LePelley ME. 2004. The role of associative history in models of associative learning: A selective review and a hybrid model. Q J Exp Psychol. 57B:192–243.

Mackintosh NJ. 1975. A theory of attention: Variations in the associability of stimuli with reinforcement. Psychol Rev. 82(4):279–298.

Nasser HM, Calu DJ, Schoenbaum G, Sharpe MJ. 2017. The Dopamine Prediction Error: Contributions to Associative Models of Reward Learning. Front Psychol. 8:244.

Papini MR, Bitterman ME. 1993. The two-test strategy in the study of inhibitory conditioning. J Exp Psychol Anim Behav Process. 19(4):342–352.

Pearce JM, Hall G. 1980. A model for Pavlovian learning: variations in the effectiveness of conditioned but not of unconditioned stimuli. Psychol Rev. 87(6):532–552.

Pears A, Parkinson JA, Hopewell L, Everitt BJ, Roberts AC. 2003. Lesions of the orbitofrontal but not medial prefrontal cortex disrupt conditioned reinforcement in primates. J Neurosci. 23(35):11189–11201.

Rescorla RA, Wagner AR. 1972. A theory of Pavlovian conditiong: Variations in the effectiveness of reinforcement and nonreinforcement. In: Black AH, Prokesy WF, editors. Classical Conditioning II: Current Research and Theory. New York: Appleton Century Crofts. p. 64–99.

Schoenbaum G, Roesch MR, Stalnaker TA, Takahashi YK. 2009. A new perspective on the role of the orbitofrontal cortex in adaptive behaviour. Nat Rev Neurosci. 10(12):885–892.

Sutton RS, Barto AG. 1998. Reinforccement Learning: An introduction. Cambridge, Massachusetts: The MIT Press.

Takahashi YK, Roesch MR, Stalnaker TA, Haney RZ, Caiu DJ, Taylor AR, Burke KA, Schoenbaum G, Calu DJ. 2009. The Orbitofrontal Cortex and Ventral Tegmental Area Are Necessary for Learning from Unexpected Outcomes. Neuron. 62(2):269–280.

Takahashi YK, Roesch MR, Wilson RC, Toreson K, O’Donnell P, Niv Y, Schoenbaum G. 2011. Expectancy-related changes in firing of dopamine neurons depend on orbitofrontal cortex. Nat Neurosci. 14(12):1590–1597.
